# Supplementary material for: Differential miRNA Expression Contributes to Emergence of Multiple Cancer Stem Cell Subpopulations in Human Colorectal Cancer
Source: J Stem Cell Res Ther. Author manuscript; Available in PMC 2024 Mar 27. (PMC10972542)
Supplement: Supplementary Material [file NIHMS1953997-supplement-Supplementary_Material.pdf]

Differential miRNA Expression Contributes to Emergence of Multiple Cancer Stem Cell Subpopulations in Human Colorectal Cancer

Victoria A. Stark [1,2] , Caroline O. B. Facey [2], Lynn Opdenaker[1,2], Jeremy Z. Fields [3], Bruce M. Boman [1,2,4,*]

[1] Department of Biological Sciences, University of Delaware, Newark, DE 19716, USA, vstark@udel.edu;

[2] Center for Translational Cancer Research, Helen F. Graham Cancer Center & Research Institute, Newark, DE 19713, USA, Caroline.Facey@christianacare.org;

[3]CATX Inc, Princeton NJ USA. JZFields@Comcast.net;

[4] Department of Pharmacology & Experimental Therapeutics, Thomas Jefferson University, Philadelphia, PA 19107, USA

*Corresponding author: Bruce M. Boman, MD, PhD
Center for Translational Cancer Research, Helen F. Graham Cancer Center & Research Institute
Department of Biological Sciences, University of Delaware
4701 Ogletown-Stanton Road, Newark, DE 19713
Phone: 267-303-9241 Email: brboman@udel.edu

Keywords: microRNAs, colorectal cancer, cancer stem cells, biomarker(s), tumor heterogeneity, ALDH, LRIG1, CD166, LGR5.

Financial support: This study was supported in part by The Lisa Dean Moseley Foundation (BB), Cancer B*Ware Foundation (BB), The Cawley Center for Translational Cancer Research Fund (VS, CF, BB), University of Delaware Department of Biological Sciences (VS), and INBRE NIH/NIGMS GM103446 (LO).

Acknowledgements: A special thanks to Dr. Erica M. Selva for her leadership of the graduate program as well as for her dedication and support of this research project. We thank Molly Lausten, Brian Osmond and Chi Zhang for helpful comments and valuable suggestions. The authors also thank Dr. Nicholas Petrelli for his support of the project that was conducted at the Center for Translational Cancer Research and we thank the Genomics Facility at the Wistar Institute for Nanostring profiling.

ABSTRACT

One reason for lack of efficacy in cancer therapeutics is tumor heterogeneity. We hypothesize that tumor heterogeneity arises due to emergence of multiple cancer stem cell (CSC) subpopulations because miRNAs regulate expression of stem cell genes in CSCs. Our goal was to determine if: *i*) multiple CSC subpopulations exist in a human CRC cell population, and *ii*) miRNAs are differentially expressed in the different CSC subpopulations. We discovered that at least four different CSC populations (ALDH1, CD166, LGR5, LRIG1) exist in the HT29 cell line. CSC subpopulations were quantified using co-staining for multiple stem cell markers, isolated using FACS, and analyzed by NanoString miRNA profiling. The miRNA expression pattern in each CSC subpopulation was analyzed relative to miRNA expression patterns in other CSC subpopulations. Messenger RNAs predicted to be targeted by the upregulated miRNAs in each CSC subpopulation were: 1) identified using bioinformatics analyses, and 2) classified according to their predicted functions using David functional annotation analyses. We found multiple CSC subpopulations with a unique miRNA signature in each CSC subpopulation. Notably, the miRNAs expressed within one CSC subpopulation are predicted to target and downregulate the CSC genes and pathways that establish the other CSC subpopulations. Moreover, mRNAs predicted to be targeted by miRNAs in the different CSC subpopulations have different cellular functional classifications. That different CSC subpopulations express miRNAs that are predicted to target CSC genes expressed in other CSC subpopulations provides a mechanism that might explain the co-existence of multiple CSC subpopulations, tumor heterogeneity, and cancer therapy resistance.

INTRODUCTION

The lack of effectiveness of many cancer treatments is attributed to tumor heterogeneity caused by co-existence of different tumor cell populations which are variably resistant to anti-cancer treatment [1,2]. We conjecture that different tumor cell populations are generated by different cancer stem cell (CSC) subpopulations, which gives rise to the tumor heterogeneity. Consequently, we have been determining whether dysregulation of miRNAs might explain how the multiple CSC subpopulations emerge in CRC. For example, our previous data showed that: *i*) miRNA23b targets the CSC gene LGR5 [3]; *ii*) miRNA92a targets the CSC gene LRIG1 [4]; *iii*) CSC genes predicted to be targeted by miRNAs correlate with reduced levels of CSC gene expression [5]; *iv*) multiple subpopulations of CSCs exist in CRCs [6]. *Hypothesis*: tumor heterogeneity arises due to emergence of multiple CSC subpopulations because specific miRNAs target different SC genes in CSCs. If each different CSC subpopulation expresses miRNAs that target SC genes expressed in other CSC subpopulations, it would provide a mechanism that might explain the emergence of CSC subpopulations and tumor heterogeneity. Accordingly, in the current study, different CSC subpopulations in the HT29 CRC cell line were 1) identified using co-staining for multiple stem cell markers, 2) isolated using fluorescence activated cell sorting (FACS), 3) analyzed by NanoString miRNA profiling of miRNA expression patterns, and 4) evaluated using bioinformatics to classify cellular function.

MATERIALS AND METHODS

Cell Culture and Maintenance

HT29 cells were purchased from American Type Culture Collection (ATCC; Manassas, VA, USA) and grown to promote a single layer. Cells were cultured in McCoy's 5A medium (GIBCO/Life Technologies) with 5% Fetal Bovine Serum (FBS) and 100 units/ml penicillin and 100 ug/ml streptomycin (1x Penicillin-Streptomycin). Cell cultures were maintained at 37 °C in humidified air at 5% CO_2. Cells were grown in T-75 flasks (flow cytometric analysis) or T-175 flasks (Fluorescence Activated Cell Sorting) (VWR International; Bridgeport, NJ, USA) every 4-5 days until confluency. If cells were passaged every week with 1 mL suspension, cells were fed fresh media by the 3-4th day. For experimentation, cells were cultured to about 50%. confluency.

ALDEFLUOR Assay

ALDEFLUOR Assay. The ALDEFLUOR assay to identify and isolate ALDH+ cells was done as we previously described [3,7]. Briefly, the ALDEFLUOR kit was purchased from Stem Cell Technologies (Cambridge, MA) and preparation of reagents were carried out according to their protocol. Cells were grown for about 2-4 days until confluency of 50% was achieved. Every 48 hours the cell culture was given fresh media. The ALDEFLUOR kit contained the following: DEAB inhibitor, DMSO, HCl, and ALDEFLUOR Assay Buffer. The ALDEFLUOR kit and its reagents were left at room temperature at the start of the protocol. For ALDEFLUOR activation, DMSO (25 uL) was added to the ALDEFLUOR Reagent. Then the tube with DMSO was mixed at room temperature for 1 minute. Next, 25 uL of 2 N HCl was added and the tube was vortexed. The tube was incubated for 15 minutes at room temperature. Lastly, ALDEFLUOR Assay Buffer (360 uL) was added to the vial and mixed. The remaining ALDEFLUOR Reagent was stored in 20 uL aliquots at -20 °C for future experiments. Note: It is essential to keep the activated ALDEFLUOR reagent at 2-8 °C on ice during use.

ALDEFLUOR test for detection of ALDH+ cells. The assay was performed according to the Stem Cell Technologies published protocol with modification. Cells were grown to 50% confluency. Adherent cells were washed with PBS and detached from the flask using 0.25% Trypsin-EDTA (Fisher Scientific). After resuspension in fresh media, a cell count was performed to calculate the appropriate amount of suspension to give 1 million cells/mL. The appropriate amount from the calculation was transferred into 2 tubes labeled as control and sample. The cells were centrifuged at 500 x G for 5 minutes to pellet and then washed with 1 mL of PBS. The process of pipetting and pelleting was repeated once more. Then the PBS was aspirated, and cells were re-suspended in 1 mL of ALDEFLUOR assay buffer. For each sample, a control tube and sample tube were prepared. To the control tube, 5 uL of the DEAB inhibitor was added. To the sample tube, 5 uL of the activated ALDEFLUOR reagent was added and mixed with 1mL of the cell/ALDEFLUOR assay buffer suspension. Immediately after vortexing, 500 uL of the suspension was removed and added to the control tube with the inhibitor. Cells were then incubated for 25 minutes at 37 °C. After incubation, cells were pelleted by centrifugation, assay buffer aspirated, and cells re-suspended in 500 uL of fresh ALDEFLUOR buffer. Cells were then passed through a Falcon round bottom tube with a 50-µm cell strainer to obtain a single cell suspension (Corning, USA). Samples were placed on ice in the dark until ready for flow cytometric analysis via the BD LSR Fortessa.

Flow Cytometry

HT29 cells were grown to 60% confluency and then lifted with Cell Stripper (Corning). After resuspension in fresh media, cells were calculated for 1 million cells per 1.5 mL tube. Cells were pelleted and then resuspended in 1 mL of Flow Cytometry Staining Buffer 1X (R&D Systems #FC001) for blocking. Cells were blocked for 1 hour on ice in the dark. After blocking, the cells were spun to aspirate the blocking buffer. The following primary antibodies were used: Anti-CD166 mouse monoclonal antibody conjugated with PE (phycoerythrin) at 5 uL (BD Biosciences #559263), anti-LRIG1 sheep polyclonal unconjugated antibody at 2.5 ug (Invitrogen #PA5-47928), and anti-LGR5 mouse monoclonal conjugated with PE at 1:100 (Origene #TA400001). Apart from the LGR5 antibody incubation (of 20 minutes), samples were incubated with antibodies for 30 minutes on ice in the dark.

The appropriate IgGs were also incorporated for the following primary antibodies. The following IgGs were included: mouse IgG1 – PE conjugate (5 uL for CD166; BD Biosciences #555749), purified sheep IgG (2 mg/mL for LRIG1 R&D Systems #5-001-A), and mouse IgG1-PE conjugate (10 uL for LGR5; R&D Systems #IC002P). It is worth noting that only the LRIG1 was incubated with a secondary antibody conjugated to allophycocyanin (APC; R&D Systems #F0127). LRIG1 cells were incubated with secondary antibody for 30 minutes in the dark on ice. Following antibody and IgG incubation, all cells were spun down to remove the antibody solution and resuspended in fresh flow cytometry staining buffer (R&D Systems #FC001). Cells were then passed through round bottom Falcon tubes for flow cytometric analysis using a BD LSR Fortessa.

Fluorescence Activated Cell Sorting (FACS)

The following protocol for FACS utilized the flow cytometry protocol for isolating the different CSC populations. The amount of buffer solution and antibody concentrations were scaled up accordingly for successful sorting of ~7 million cells where two populations were analyzed. Once the protocol was completed, all cells were passed through the Falcon round

bottom tube for FACS analysis via the BD FACS Aria II. After sorting, cells were spun down to remove supernatant. Next, 500 uL of Trizol was added to each tube and vortexed for 20 seconds. Lastly the tubes were set at room temperature in Trizol for 10 minutes. Following room temperature incubation, samples were transferred into 1.5 mL tubes, labeled, and stored at -80° C. For the combination sorts that involved the LGR5 antibody, sorted cells were centrifuged and placed in RNA Lysis Buffer from Zymo Research. The tubes were then immediately placed into the -80° C freezer for storage. Samples remained in storage until each set with appropriate replicates were complete and ready to be sent for NanoString miRNA profiling at the Wistar Genomics Facility (Philadelphia, PA).

miRNA Profiling

The data received from the Genomics Core Facility at the Wistar Institute was then analyzed using the nSolver 4.0 from NanoString Technologies (Seattle, WA). Heat maps were generated for the TOP 50 miRNAs expressed for each of the different sort combinations of LRIG1, LGR5, CD166, and ALDH. For clarification, the sort combinations were done in pairs of two during FACS and NanoString profiling analysis. We then generated a list of ratios for each pair of stem cell subpopulations and sorted the list according to their respective *p*-values. Only statistically significant ($p<0.05$) differentially expressed miRNAs were selected. The first 50 miRNAs with the highest expression levels were selected and used to generate the heatmaps presented in this paper.

Statistical Analysis

T-tests and its corresponding p-values for the different miRNAs were generated by the nSolver 4.0 software. The interpretation of the different miRNA p-values helped designate our confidence in the miRNAs that are said to target LRIG1, CD166, LGR5, and ALDH, respectively.

Bioinformatics Analysis

Bioinformatics analysis was done using the TCGA database [Pan, C-Y and Lin, W-C; miR-TV Introduction available online] and miRbase.org to look for individual miRNAs and the relevant clusters that are targeted in stem cell genes. In addition to the targeted SC genes, we analyzed the miRNA expression level and different clinical correlations with CRC. Lastly, we also investigated miRNA isoform (isomiR) expression as it displayed variability in expression regarding the cancer type. Additional databases used were: i) miRBase for identifying miRNA clusters [miRbase Available online], TargetScan for identifying isomiRs and predicted miRNA targets [TargetScanHuman 7.2 Available online], and GeneCards for identifying miRTarBase predicted miRNA targets [GeneCards – Human Genes | Gene Database | Gene Search Available online]. The DAVID functional annotation tool was used to classify the different mRNA functions.

<u>RESULTS</u>

Our approach was to use flow cytometric analysis to determine the proportion of different CSC subpopulations in the HT29 CRC cell line. The bar graph shown in Figure 1 gives the percentage of CSCs in the HT29 CRC cell population that stain for the different SC markers: ALDH+ (59.3%), LRIG1+ (40.2%), CD166+ (25.2%), and LGR5+ (10.2%) cells. This analysis

raised the question: Can specific CSC subpopulations be identified by analyses for the expression of multiple different SC markers?

We then evaluated six different combinations of SC marker pairs: CD166 & ALDH, LRIG1 & CD166, LRIG1 & ALDH, LGR5 & LRIG1, LGR5 & CD166, LGR5 & ALDH (described in Materials & Methods). Thus, in each of the different experiments, co-staining for two different SC markers was analyzed which led to the isolation of four different cell subpopulations: cells that are co-positive for both markers (double positive), cells that are positive for just one marker or the other marker (two different populations positive for a single marker), and cells that are negative for both markers (double negative). The miRNA expression in each cell subpopulation was then analyzed using NanoString profiling to identify miRNAs that are differentially expressed in the different CSC subpopulations (Table S1). The genes (mRNAs) that were predicted to be targeted by differentially expressed miRNAs in each CSC subpopulation were then identified, and the function of these mRNAs was classified using bioinformatics analysis (see Methods).

Results from the LGR5 and ALDH sort are presented in Figure 2. The results on the remaining CSC subpopulations are in the supplementary data section (Supplemental Figures S1-S5). The different functional classifications of the mRNAs predicted to be targeted by the miRNAs in each cell subpopulation are given in Table S2. We also did a more detailed analysis of the results from the LGR5 & ALDH sort because it had the lowest size (<1%) of the co-positive (LGR5+/ALDH+) cell subpopulation, and one of the highest numbers of differentially expressed miRNAs (Table S1). In view of underlying mechanisms, these findings might reflect to differences in functionality between LGR5+ cells and ALDH+ cells and the function of the genes that are targeted by miRNAs in the different subpopulations.

The proportions of the different cell subpopulations (LGR5+/ALDH–, ALDH+/LGR5–, LGR5+/ALDH+, LGR5–/ALDH–) are shown as a FACS dot plot and bar graph in Figures 2A & 2B. Differential expression of the miRNAs is shown as a heatmap in Figures 2C & 2D. The top 10 miRNAs as ranked by *p*-value in the LGR5+/ALDH– subpopulation and in the ALDH+/LGR5– subpopulation are listed in Figures 3 & 4, respectively. The mRNAs predicted to be targeted by these miRNAs were identified using bioinformatics analysis and the functional classification of these mRNAs is provided in the pie chart graphs in Figures 3 & 4. The functional classification of mRNAs predicted to be targeted by miRNAs identified in LGR5+/ALDH– cells was transcriptional regulation and zinc finger motifs (Figure 3). In contrast, the classification of predicted mRNAs in ALDH+/LGR5– cells was phosphoproteins and protein binding (Figure 4).

We further analyzed results from the LGR5 & ALDH sort to determine what might distinguish the different CSC subpopulations from one another. We did this using two different approaches. First, we analyzed the top 10 miRNAs ranked based on level of upregulation of miRNA expression in the LGR5+/ALDH– subpopulation versus ALDH+/LGR5– subpopulation. Bioinformatics analysis was then used to determine which genes in the RA and WNT signaling pathways (Tables S2 & S3) were predicted to be targeted by these miRNAs. We found that 35 mRNAs in the RA signaling pathway are predicted to be targeted by the top miRNAs expressed in LGR5+/ALDH– cells (Figure 4) and 18 mRNAs in the WNT signaling pathway are predicted to be targeted by the top miRNAs expressed in ALDH+/LGR5– cells (Figure 5).

In our second approach, we analyzed miRNAs that are selectively expressed in each CSC subpopulation. Specifically, we analyzed miRNAs that are expressed in LGR5+/ALDH– cells but not expressed in ALDH+/LGR5– cells, and vice versa. This analysis showed that in the RA

signaling pathway, 25 mRNAs (mostly dehydrogenases) are predicted to be targeted by miRNAs selectively expressed in LGR5+/ALDH– cells. We also found that 2 mRNAs in the WNT signaling pathway are predicted to be targeted by miRNAs selectively expressed in ALDH+/LGR5– cells. The miRNAs that were predicted to selectively target LRP5/6, AXIN2, and the dehydrogenases from ALDH & LGR5 experiment were further analyzed to see if these miRNAs are expressed in CSC subpopulations isolated from our other FACS sorting experiments (Table 2). Indeed, we identified an miRNA signature for ALDH+ cells (miR-16-5p, miR-23a-3p, miR15b-5p, miR-15a-5p, miR375, miR107), and for LGR5+ cells (miR-4521, miR-630, miR-1322, miR-519b-5p, miR-4516, miR-1285-3p, miR-1289, miR-495-5p). Thus, based on miRNA expression in CSC subpopulations from our other FACS sorts, we found that there are specific miRNA signatures that characterize ALDH+ cells and LGR5+ cells.

DISCUSSION

The key findings in our study are: 1) miRNAs are selectively expressed in different CSC subpopulations; 2) miRNAs that are predicted to target and downregulate genes that encode CSC proteins in one CSC subpopulation are SC genes likely upregulated in the other CSC subpopulation. These findings support our hypothesis that tumor heterogeneity arises due to emergence of multiple CSC subpopulations because specific miRNAs target different SC genes in CSCs.

Using flow cytometry and FACS analyses, we found that four different CSC subpopulations were present in each experiment: two single CSC populations, a co-positive and a co-negative population. The presence of 4 subpopulations shows that intra-tumoral CSC heterogeneity exists in a CRC cell line. We found that, among the different experiments, the LGR5 & ALDH sort had the lowest co-positive staining, indicating that LGR5 and ALDH mark distinctly different CSC subpopulations.

We next determined, using NanoString profiling, which miRNAs are differentially expressed between the different CSC subpopulations, and identified mRNAs predicted to be targeted by the upregulated miRNAs using additional bioinformatics tools. Notably, a unique miRNA signature was identified for each CSC subpopulation. The mRNAs predicted to be targeted by the miRNAs in each CSC subpopulation were found to have very different functional classifications (Table S2). Notably, bioinformatics analyses of NanoString profiling results showed that many targeted mRNAs play important functional roles in maintaining properties of stemness. For example, the miRNAs that are up in the ALDH+/LGR5– subpopulation are predicted to target and suppress expression of genes in WNT signaling. And, these WNT pathway genes are predicted to be upregulated in the LGR5+/ALDH– subpopulation. Conversely, the miRNAs that are up in LGR5+/ALDH– subpopulation are predicted to target and downregulate expression of genes (dehydrogenases) involved in retinoic acid (RA) signaling. And, these RA pathway genes are predicted to be upregulated in the ALDH+/LGR5– subpopulation. Thus, our results indicate that the miRNAs expressed in LGR5+/ALDH– CSCs decrease the expression of mRNAs that encode proteins essential for the existence of ALDH+/LGR5– CSCs, and vice versa.

Components of RA signaling were evaluated because RA signaling appears to mainly occur through ALDH+ SCs [7,8]. Indeed, ALDH is a key component in RA pathway and ALDH is a key SC marker that can track CSC overpopulation during CRC development [9]. Components of WNT signaling were also considered because over 90% of CRC patients have mutations in the WNT pathway [10]. It is constitutively activated WNT signaling, due to *APC*

mutations, that is the main driver of CRC growth and development [11,12]. The WNT signaling pathway is also important as LGR5 is a receptor for R-spondins and is a key factor in the canonical WNT signaling pathway [13]. Notably, the WNT pathway incorporates signaling via LGR5 in CRC growth. Accordingly, we evaluated the different mRNAs in the WNT and RA signaling pathways that are predicted to be targeted by top miRNAs in LGR5+/ALDH– cells and in ALDH+/LGR5– cells.

Thus, investigating the role of miRNAs in regulation of RA and WNT signaling is a logical step to further understand how dysregulated miRNAs contribute to emergence of different CSC subpopulations. Accordingly, we took a bioinformatics approach to investigate predicted functions for the mRNAs targeted by the miRNAs in LGR5+/ALDH– and ALDH+/LGR5– cells. We surmised that RA and WNT signaling pathways are likely downregulated by miRNAs in LGR5+/ALDH– and ALDH+/LGR5– cells, respectively.

In our bioinformatics analysis of miRNAs in LGR5+/ALDH– cells, we discovered that many of these miRNAs are predicted to target expression of a number of dehydrogenases in the RA signaling pathway. These dehydrogenases included aldehyde dehydrogenase (ALDH), alcohol dehydrogenase (ADH), and retinol dehydrogenase (RDH). While LGR5 plays a key role in WNT signaling, its role in RA signaling is largely unknown. However, our previous studies indicated that the WNT and RA signaling pathways are linked and play a role in CRC development [14]. Thus, it is possible that RA signaling and WNT signaling are inversely correlated to each other, and have opposing functions that maintain co-existence of colonic SC populations and homeostasis of colon tissues. Other studies show RA receptors can induce or downregulate WNT/β-catenin signaling during chondrocyte development [15] and development of cardiac/skeletal muscle [16]. RA signaling and WNT signaling also have opposing functional roles that control the development of different parts of the embryo [17,18].

In our bioinformatics analysis of miRNAs in ALDH+/LGR5– cells, we discovered that many of these miRNAs are predicted to target expression of AXIN2 and LRP5, which are components of the WNT signaling pathway. The WNT pathway mRNAs targets were of particular interest to evaluate because LGR5 is known to promote WNT/β-catenin signaling in the SC origin of CRCs [19]. In CRC, LRP5/6 plays an important role in WNT signaling as the WNT ligands bind to LRP5/6 and Frizzled receptors to promote WNT signaling via other WNT pathway components such as AXIN2 and β-catenin [20]. Indeed, AXIN2 is classified as a tumor suppressor gene, and AXIN2 germline mutations occur in hereditary CRC patients and predispose them to develop CRC [21]. Moreover, as noted above, LRP5 acts as a receptor for WNT ligands [20]. Thus, our findings indicate that cross regulatory miRNA-based mechanisms control expression of WNT and RA signaling components that leads to emergence of different CSC subpopulations in CRC tissues.

CONCLUSIONS

Our results provide key information on a mechanism that explains how miRNA expression plays a role in the emergence of multiple CSC subpopulations that contributes to tumor heterogeneity during CRC development. Specifically, our findings indicate that multiple CSC subpopulations emerge in HT29 CRC cells due to the expression of unique miRNAs that target different SC genes (and their co-expressed genes), resulting in intra-tumoral CSC heterogeneity. Thus, our research study has vast clinical significance because it provides insight into how dysregulation of miRNA expression leads to emergence of CSC subpopulations in CRC, which advances our understanding of what causes tumor heterogeneity. Our findings also

provide clues as to how to use miRNA-based drugs to specifically target and eliminate CSCs and improve efficacy of anti-cancer therapies [22-25].

REFERENCES

1. Fisher R, Pusztai L, Swanton C. Cancer heterogeneity: implications for targeted therapeutics. *Br J Cancer* 2013;108:479-485.

2. Blank A, Roberts DE 2nd, Dawson H, Zlobec I, Lugli A. Tumor Heterogeneity in Primary Colorectal Cancer and Corresponding Metastases. Does the Apple Fall Far From the Tree? *Front Med* (Lausanne). 2018; 5:234. doi: 10.3389/fmed.2018.00234.

3. Viswanathan V, Damle S, Zhang T, Opdenaker L, Modarai S, Accerbi M, Schmidt S, Green P, Galileo D, Palazzo J, Fields J, Haghighat S, Rigoutsos I, Gonye G, Boman BM. An miRNA Expression Signature for the Human Colonic Stem Cell Niche Distinguishes Malignant from Normal Epithelia. *Cancer Res* 2017; 77:3778-3790. doi: 10.1158/0008-5472.CAN-16-2388.

4. Viswanathan V, Opdenaker L, Modarai S, Fields JZ, Gonye G, Boman BM. MicroRNA Expression Profiling of Normal and Malignant Human Colonic Stem Cells Identifies miRNA92a as a Regulator of the LRIG1 Stem Cell Gene. *Int J Mol Sci* 2020;21:2804. doi: 10.3390/ijms21082804.

5. Stark VA, Facey COB, Viswanathan V, Boman BM. The Role of miRNAs, miRNA Clusters, and isomiRs in Development of Cancer Stem Cell Populations in Colorectal Cancer. *Int J Mol Sci* 2021; 22:1424. doi: 10.3390/ijms22031424.

6. Viswanathan, V. MicroRNAs in Normal and Malignant Colon Stem Cells and Their Possible Role in Stem Cell Origin of Colon Cancer. Ph.D. Dissertation, 2014; pp.1–169. http://udspace.udel.edu/handle/19716/16788 (accessed on July 8, 2021).

7. Modarai SR, Gupta A, Opdenaker LM, Kowash R, Masters G, Viswanathan V, Zhang T, Fields JZ, Boman BM. The anti-cancer effect of retinoic acid signaling in CRC occurs via decreased growth of ALDH+ colon cancer stem cells and increased differentiation of stem cells. *Oncotarget* 2018; 9:34658-34669. doi: 10.18632/oncotarget.26157.

8. Hunsu VO, Facey COB, Fields JZ, Boman BM. Retinoids as Chemo-Preventive and Molecular-Targeted Anti-Cancer Therapies. *Int J Mol Sci* 2021;22:7731. doi: 10.3390/ijms22147731.

9. Huang EH, Hynes MJ, Zhang T, Ginestier C, Dontu G, Appelman H, Fields JZ, Wicha MS, Boman BM. Aldehyde dehydrogenase 1 is a marker for normal and malignant human colonic stem cells (SC) and tracks SC overpopulation during colon tumorigenesis. *Cancer Res* 2009; 69:3382-9. doi: 10.1158/0008-5472.CAN-08-4418.

10. Cancer Genome Atlas Network. Comprehensive molecular characterization of human colon and rectal cancer. *Nature* 2012; 487:330-7. doi: 10.1038/nature11252.

11. Prosperi JR, Luu HH, Goss KH. Dysregulation of the Wnt pathway in solid tumors. In Targeting the Wnt Pathway in Cancer; Springer: New York, NY, USA, 2011.

12. Saito-Diaz K, Chen TW, Wang X, Thorne CA, Wallace HA, Page-McCaw A, Lee E. The way Wnt works: components and mechanism. Growth Factors. 2013; 31:1-31. doi: 10.3109/08977194.2012.752737.

13. Barker N, van Es JH, Kuipers J, Kujala P, van den Born M, Cozijnsen M, Haegebarth A, Korving J, Begthel H, Peters PJ, Clevers H. Identification of stem cells in small intestine and colon by marker gene Lgr5. *Nature*. 2007; 449:1003-7. doi: 10.1038/nature06196.

14. Zhang T, Ahn K, Emerick B, Modarai SR, Opdenaker LM, Palazzo J, Schleiniger G, Fields JZ, Boman BM. *APC* mutations in human colon lead to decreased neuroendocrine maturation of ALDH+ stem cells that alters GLP-2 and SST feedback signaling: Clue to a link between WNT and retinoic acid signalling in colon cancer development. *PLoS ONE* 2020 15:e0239601. https://doi.org/10.1371/journal.pone.0239601.

15. Yasuhara R, Yuasa T, Williams JA, Byers SW, Shah S, Pacifici M, Iwamoto M, Enomoto-Iwamoto M. Wnt/beta-catenin and retinoic acid receptor signaling pathways interact to regulate chondrocyte function and matrix turnover. *J Biol Chem* 2010; 285:317-27. doi: 10.1074/jbc.M109.053926.

16. Kennedy, K.A.M *et al.* (2009). Retinoic acid enhances skeletal muscle progenitor formation and bypasses inhibition by bone morphogenetic protein 4 but not dominant negative β-catenin. BMC biology. 7. 67. 10.1186/1741-7007-7-67.

17. Zhao X, Duester G. Effect of retinoic acid signaling on Wnt/beta-catenin and FGF signaling during body axis extension. *Gene Expr Patterns* 2009; 9:430-5. doi: 10.1016/j.gep.2009.06.003.

18. Wang S, Huang H, Xiang H, Gu B, Li W, Chen L, Zhang M. Wnt Signaling Modulates Routes of Retinoic Acid-Induced Differentiation of Embryonic Stem Cells. *Stem Cells Dev* 2019; 28:1334-1345. doi: 10.1089/scd.2019.0065.

19. Barker N, Ridgway RA, van Es JH, van de Wetering M, Begthel H, van den Born M, Danenberg E, Clarke AR, Sansom OJ, Clevers H. Crypt stem cells as the cells-of-origin of intestinal cancer. *Nature* 2009; 457:608-11.

20. Saito-Diaz K, Chen TW, Wang X, Thorne CA, Wallace HA, Page-McCaw A, Lee E. The way Wnt works: components and mechanism. *Growth Factors* 2013; 31:1-31. doi: 10.3109/08977194.2012.752737.

21. Mazzoni SM, Fearon ER. AXIN1 and AXIN2 variants in gastrointestinal cancers. *Cancer Lett* 2014; 355:1-8. doi: 10.1016/j.canlet.2014.09.018.

22. Ma YS, Li W, Liu Y, Shi Y, Lin QL, Fu D. Targeting Colorectal Cancer Stem Cells as an Effective Treatment for Colorectal Cancer. *Technol Cancer Res Treat* 2020; 19:1533033819892261. doi: 10.1177/1533033819892261.

23. Facey COB, Boman BM. Retinoids in Treatment of Colorectal Cancer [Online First], IntechOpen 2020, doi: 10.5772/intechopen.93699.

24. Morgan RG, Mortensson E, Williams AC. Targeting LGR5 in Colorectal Cancer: therapeutic gold or too plastic? *Br J Cancer* 2018; 118:1410-1418. doi: 10.1038/s41416-018-0118-6.

25. Viswanathan V, Fields J, Boman BM The *miRNA23b*-regulated signaling network as a key to cancer development – implications for translational research and therapeutics. *J Mol Medicine* 2014; 92:1129-38. doi: 10.1007/s00109-014-1208-4.

26. Si W, Shen J, Zheng H, Fan W. The role and mechanisms of action of microRNAs in cancer drug resistance. *Clin Epigenet* 2019; 11:25. doi: 10.1186/s13148-018-0587-8.

FIGURE LEGENDS

Figure 1. Proportion of different CSC subpopulations in the HT29 CRC cell line. The bar graph gives the percent ALDH+, LRIG1+, CD166+, and LGR5+ cells in the HT29 CRC cell population as determined by flow cytometric analysis. Error bars represent standard error of the mean (n = 3-7 experimental replicates per marker).

Figure 2.Proportion of different CSC subpopulations from FACS analysis of LGR5 and ALDH marker expression. This figure gives results from FACS analysis of stained CRC cells using a combination of the SC marker pair ALDH & LGR5. *Panel A* gives a representative dot blot graph illustrating the proportion of cells in LGR5+/ALDH–, ALDH+/LGR5–, LGR5+/ALDH+, and LGR5–/ALDH– cell subpopulations. *Panel B* gives a bar graph showing the average percentage positive cells in each of the four different CSC subpopulations. Error bars represent standard error of the mean. *Panel C* gives a heatmap from NanoString profiling analysis showing differential expression of the top 50 miRNAs in the four different isolated CSC subpopulations (increased expression = green; decreased expression = red for each CSC marker). NanoString miRNA profiling. *Panel D* provides the same heatmap with the respective dendrogram. Panels C and D visually illustrate the patterns that are seen when a large set of miRNAs is surveyed and they are not meant to show details, which is why the vertical axes are not legible.

Figure 3. The top 10 miRNAs and functional analysis of predicted targeted genes in LGR5+/ALDH– cells. The table lists the top 10 miRNAs expressed in LGR5+/ALDH– cells. miRNAs were ranked according to p-value <0.05 for statistical significance. The asterisk depicts the original name for the miRNA that was abbreviated in the Table. The pie chart shows the functional classification identified by David analysis of the mRNAs predicted to be targeted by the miRNAs expressed in LGR5+/ALDH– cells. A summary of the functions of mRNAs predicted to be targeted by the miRNAs is given in Table S2.

Figure 4. The top 10 miRNAs and functional analysis of predicted targeted genes in ALDH+/LGR5– cells. The table lists the top 10 miRNAs expressed in ALDH+/LGR5– cells. miRNAs were ranked according to p-value <0.05 for statistical significance. The pie chart shows the functional classification identified by David analysis of the mRNAs predicted to be targeted by the miRNAs expressed in ALDH+/LGR5– cells. A summary of the functions of mRNAs predicted to be targeted by the miRNAs is given in Table S2.

Figure 5. A string network map of genes in the retinoic acid signaling pathway that are predicted to be targeted by miRNAs expressed in LGR5+/ALDH– cells. The following 25 mRNAs are predicted to be targeted by miRNAs that are selectively expressed in LGR5+ cells (not expressed in ALDH+ cells): *ADH1B, ADH4, ADH5, ADH6, ADH7, ALDH16A1, ALDH1A3, ALDH1L1, ALDH2, ALDH3A1, ALDH3A2, ALDH3B2, ALDH4A1, ALDH5A1, ALDH7A1, ALDH9A1, CRABP2, RBP2, RBP4, RBP5, RDH11, RDH13, RDH5, RDH8, RXRB.*

Figure 6. A string network map of genes in the WNT signaling pathway that are predicted to be targeted by miRNAs expressed in ALDH+/LGR5– cells. Two mRNAs in the WNT signaling pathway (*AXIN2* & *LRP5*) are predicted to be targeted by miRNAs selectively expressed in ALDH+ cells (not expressed in LGR5+ cells).

SUPPLEMENTAL FIGURE LEGENDS

Figures S1-S5 give results from FACS isolation and NanoString profiling analysis of stained CRC cells using five different combinations of SC marker pairs: S1) CD166 & ALDH, S2) LRIG1 & CD166, S3) LRIG1 & ALDH, S4) LGR5 & LRIG1, S5) LGR5 & CD166. In each figure, *Panel A* gives a FACS dot blot graph from a representative experiment showing proportion of the cells in the four different cell subpopulations: cells that are co-positive for both markers (double positive), cells that are positive for just one marker or the other marker (two different populations positive for a single marker), and cells that are negative for both markers (double negative). *Panel B* gives a bar graph that shows the average percentage positive cells in each of these four different CSC subpopulations. Error bars represent standard error of the mean. *Panel C* gives a heatmap showing differential expression of the top 50 miRNAs in the four different isolated CSC subpopulations (increased expression = green; decreased expression = red for each CSC marker). *Panel D* shows the same heatmap with the corresponding dendrogram. Panels C and D visually illustrate the patterns that are seen when a large set of miRNAs is surveyed and they are not meant to show details, which is why the vertical axes are not legible. *Panel E* gives a table listing the top 10 miRNAs expressed in each subpopulation. *Panel F* gives a pie chart graph showing the functional classification identified by David analysis of the mRNAs predicted to be targeted by the miRNAs expressed in each subpopulation. In each analysis, the top 10 miRNAs were ranked by *p*-value ($p < 0.05$) in order to determine the mRNAs that are predicted to be targeted by miRNAs in each subpopulation. Table S2 gives a summary of the functions of mRNAs predicted to be targeted by the miRNAs.

Table 1. MicroRNA expression patterns in ALDH+ cells and LGR5+ cells in CSC sub-populations isolated from different FACS sorting experiments

	**LGR5/ALDH Sort**	**LGR5/CD166 Sort**	**LGR5/LRIG1 Sort**	**ALDH/LRIG1 Sort**
	**ALDH+ Cells**	**LGR5+ Cells**	**LGR5+ Cells**	**ALDH+ Cells**
miR-16-5p	↑	↓	↓	↑
miR-23a-3p	↑	↓	↓	↑
miR-15b-5p	↑	↓	↓	↑
miR-15a-5p	↑	↓	↓	↑
miR-375	↑	↓	↑	↑
miR-107	↑	↑	↓	↑
	**LGR5+ Cells**	**LGR5+ Cells**	**LGR5+ Cells**	**ALDH+ Cells**
miR-4521	↑	↑	↑	↓
miR-630	↑	↑	↑	↓
miR-1322	↑	↑	↑	↓
miR-519b-5p	↑	↑	↑	↓
miR-4516	↑	↑	↑	↓
miR-1285-3p	↑	↑	↑	↓
miR-1289	↑	↑	↑	—
miR-495-5p	↑	↑	↑	↓

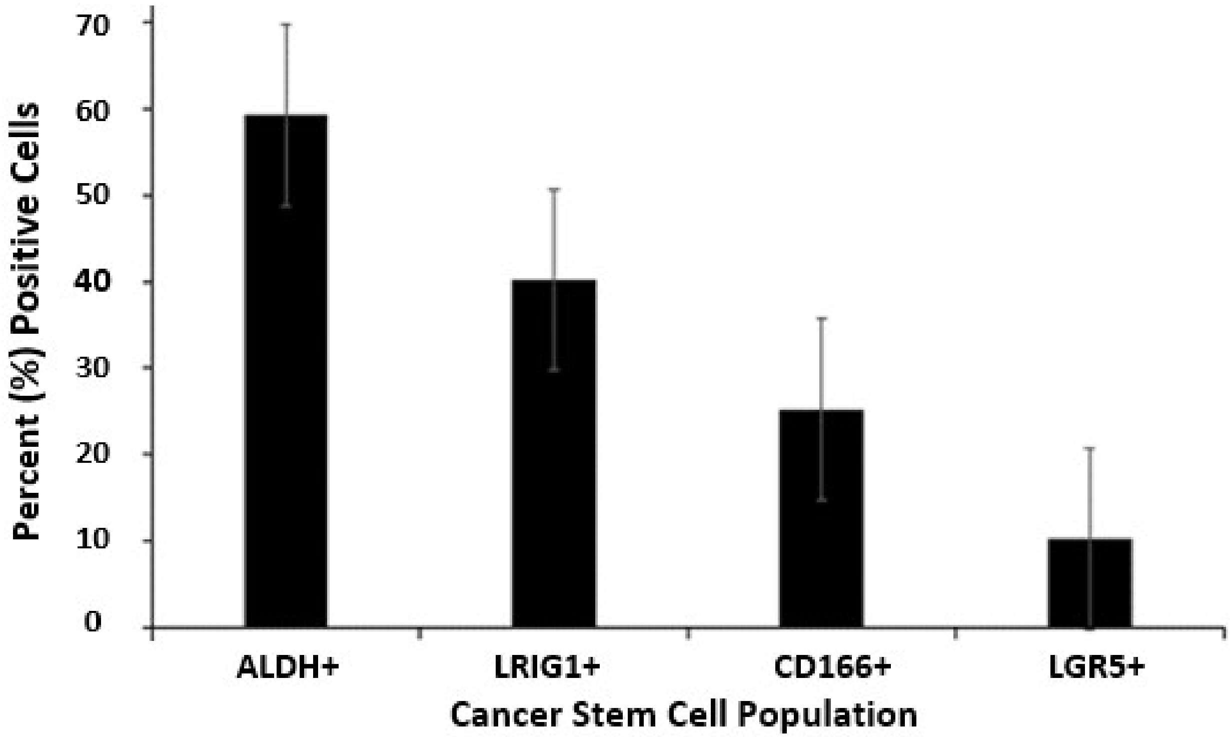
70
60
50
40
30
20
10
0
Percent (%) Positive Cells
ALDH+
LRIG1+
CD166+
LGR5+
Cancer Stem Cell Population

Figure 1

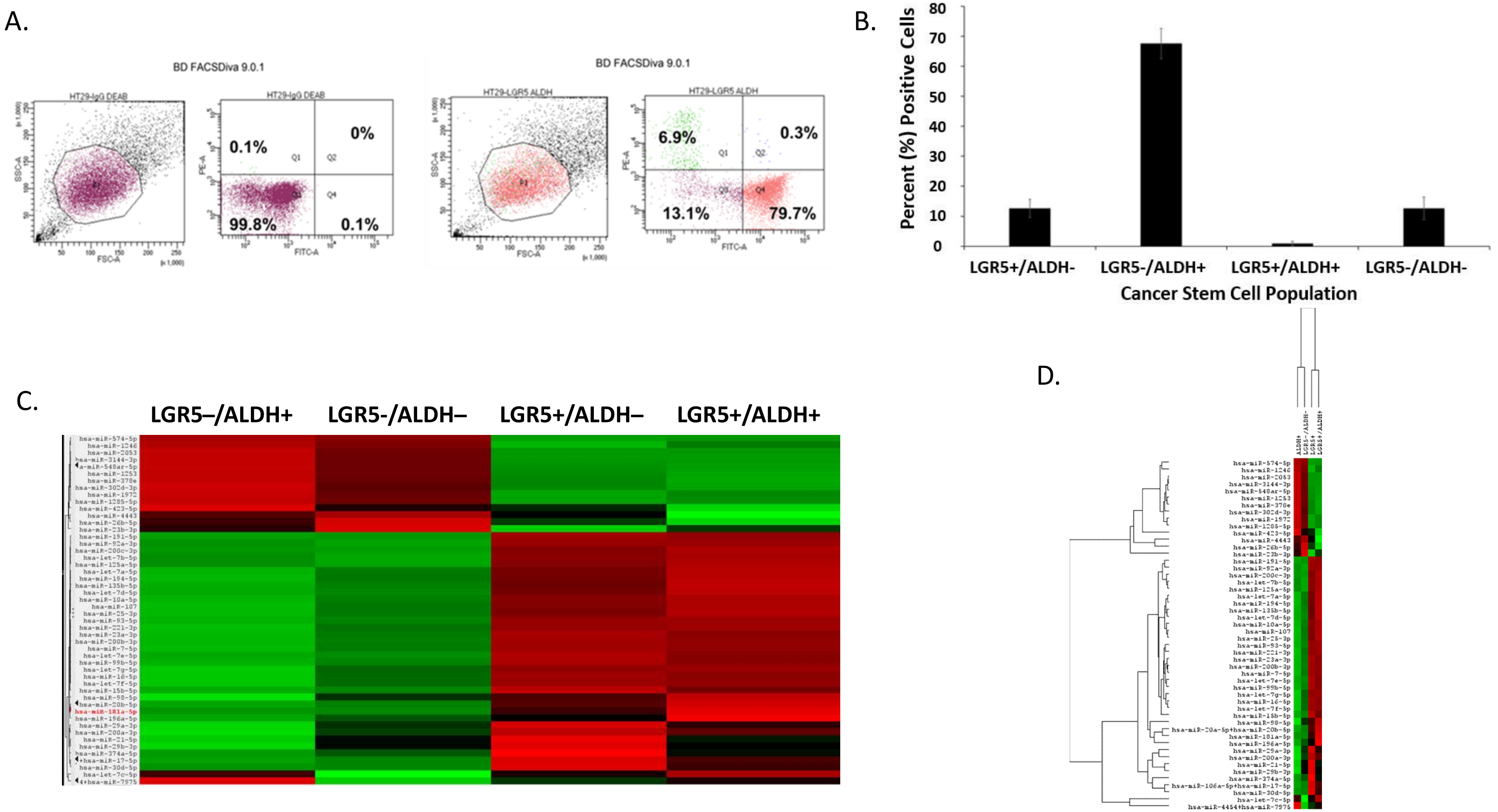
A.
BD FACSDiva 9.0.1
HT29-IgG DEAB
0.1%
0%
99.8%
0.1%
BD FACSDiva 9.0.1
HT29-LGR5 ALDH
6.9%
0.3%
13.1%
79.7%
B.
Percent (%) Positive Cells
80
70
60
50
40
30
20
10
0
LGR5+/ALDH-
LGR5-/ALDH+
LGR5+/ALDH+
LGR5-/ALDH-
Cancer Stem Cell Population
C.
LGR5–/ALDH+
LGR5-/ALDH–
LGR5+/ALDH–
LGR5+/ALDH+
D.

Figure 2

LGR5+/ALDH-Top miRNAs

Name	*p*-Value	Ratio
hsa-miR-4521	0.0028	7.38
hsa-miR-630	0.003023	7.23
hsa-miR-1322	0.003876	6.57
hsa-miR-663a	0.004073	8.14
hsa-miR-519b-5p*	0.00447	7.83
hsa-miR-4516	0.004558	10.20
hsa-miR-30e-5p	0.004601	5.01
hsa-miR-1285-3p	0.004601	6.81
hsa-miR-1289	0.004761	6.31
hsa-miR-495-5p	0.004805	8.41

*hsa-miR-519b-5p+hsa-miR-519c-5p+hsa-miR-523-5p+hsa-miR-518e-5p+hsa-miR-522-5p+hsa-miR-519a-5p

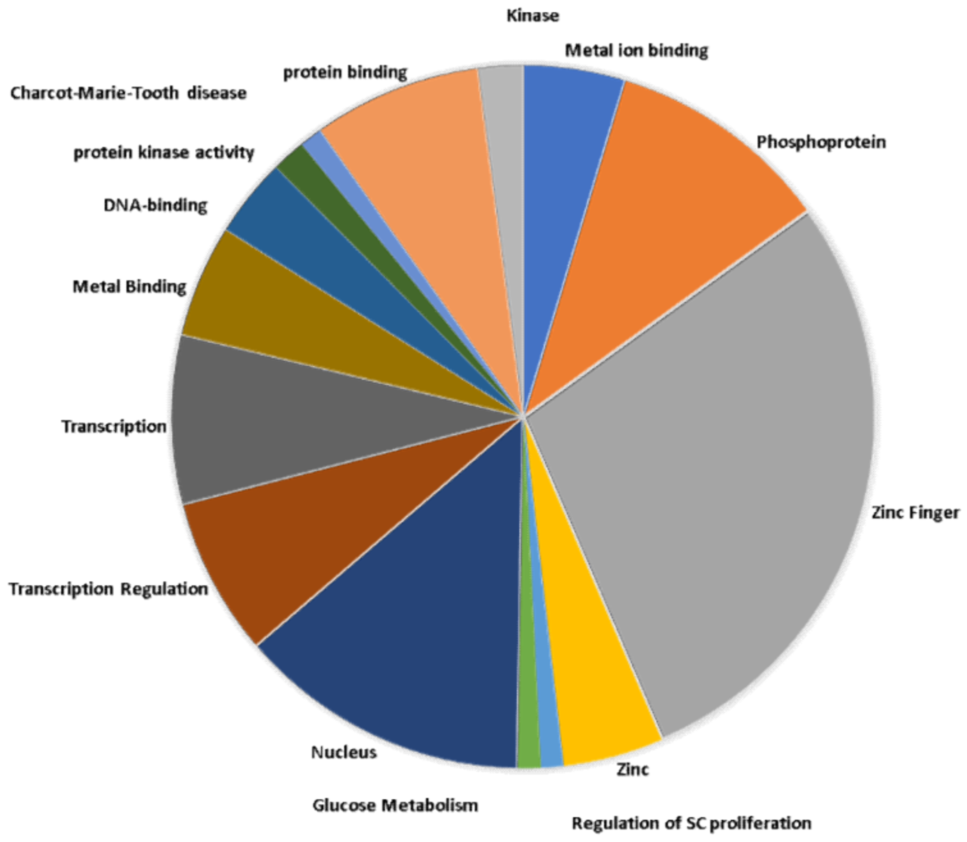

Figure 3

ALDH+/LGR5- Top miRNAs

Name	*p*-Value	Ratio
hsa-miR-194-5p	0.007798	5.88
hsa-miR-7-5p	0.008939	2.86
hsa-miR-16-5p	0.011747	3.57
hsa-let-7g-5p	0.012033	2.86
hsa-miR-182-5p	0.012382	2.63
hsa-miR-19a-3p	0.013906	5.00
hsa-miR-23a-3p	0.014909	2.50
hsa-miR-15b-5p	0.0174	5.26
hsa-miR-99b-5p	0.02062	2.38
hsa-miR-200b-3p	0.025989	4.17

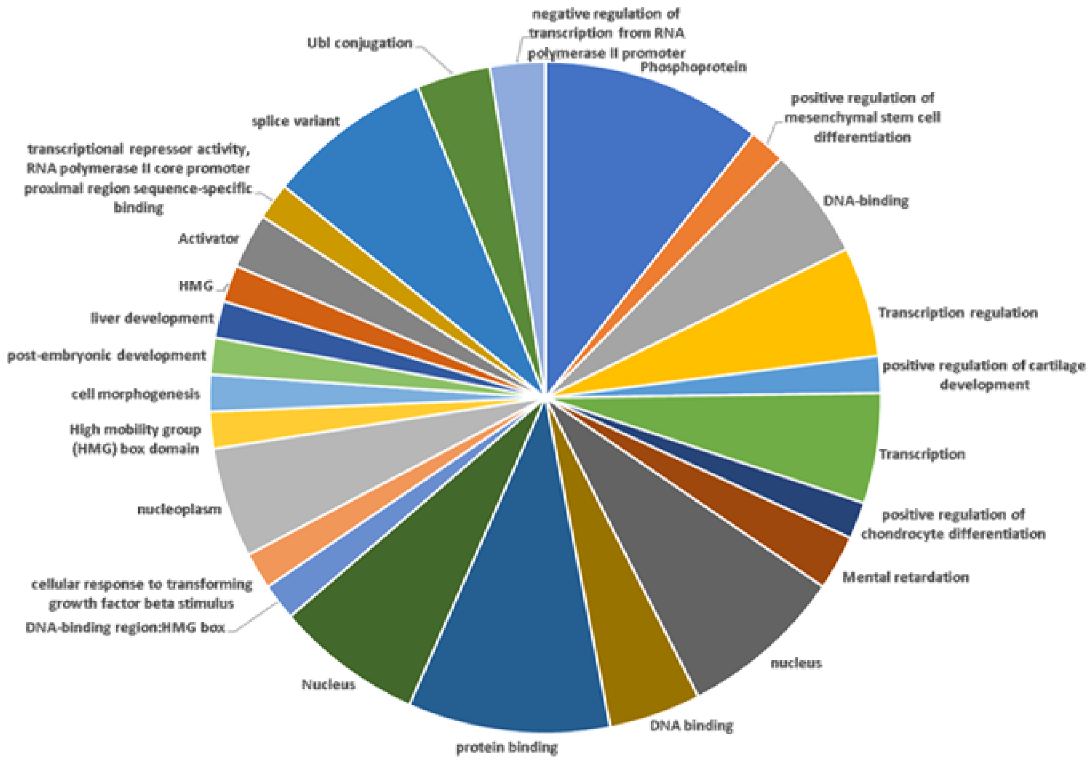

Figure 4

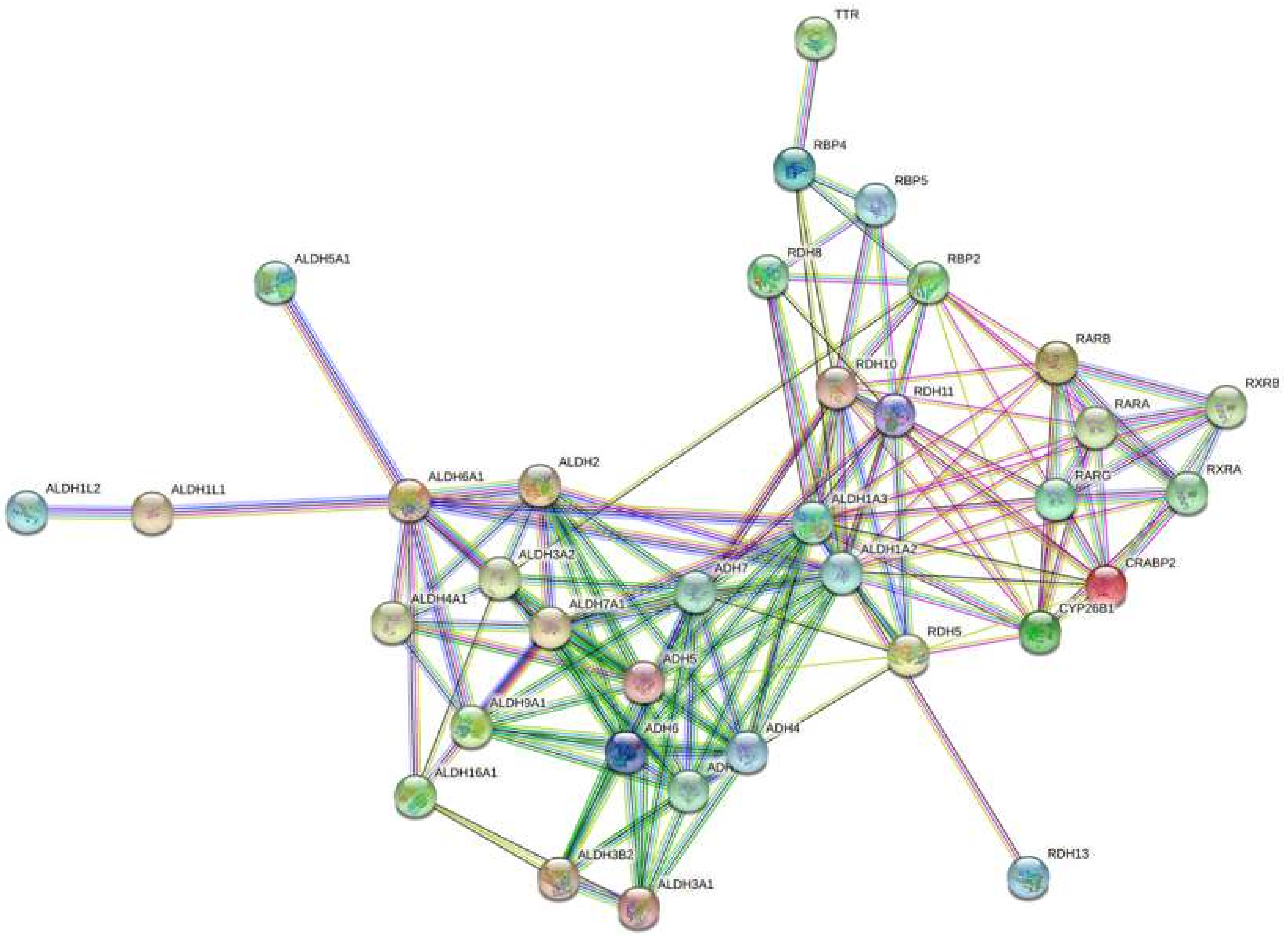
TTR
RBP4
RBP5
RDH8
RBP2
ALDH5A1
RARB
RDH10
RDH11
RXRB
RARA
ALDH2
ALDH6A1
ALDH1L2
ALDH1L1
RARG
RXRA
ALDH1A3
ALDH1A2
CRABP2
ALDH3A2
ADH7
ALDH4A1
ALDH7A1
CYP26B1
RDH5
ADH5
ALDH9A1
ADH6
ADH4
ALDH16A1
RDH13
ALDH3B2
ALDH3A1

Figure 5

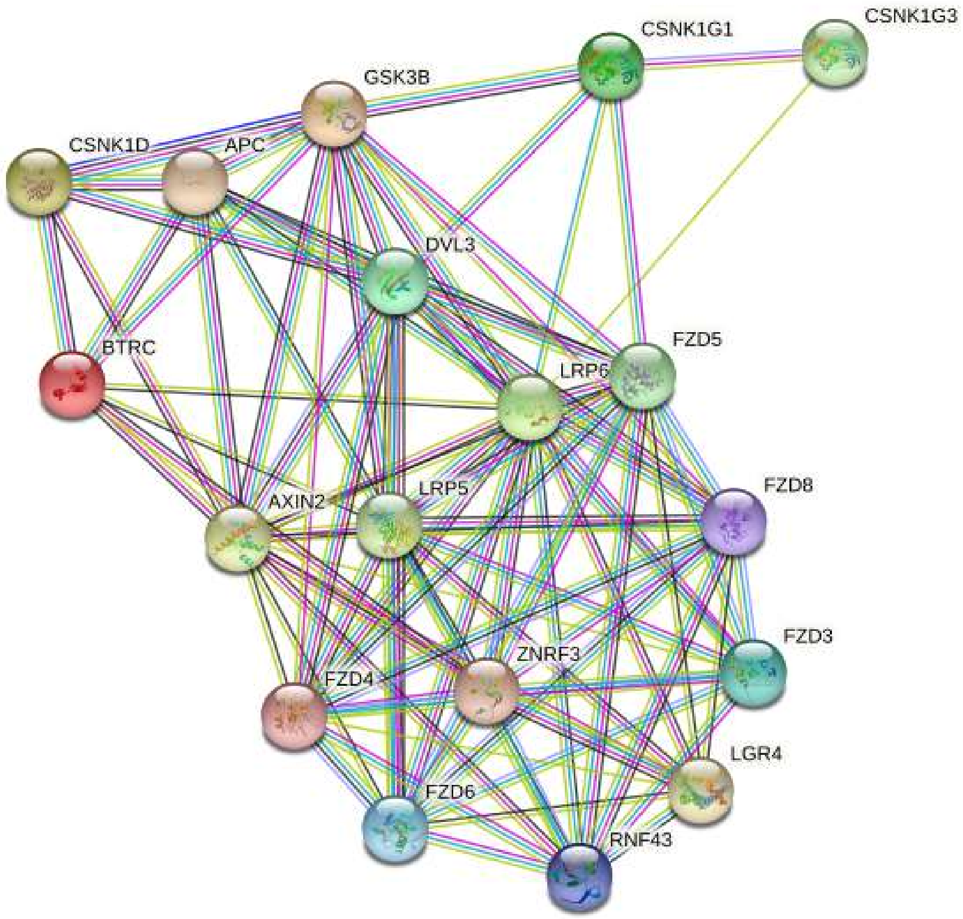
CSNK1G1
CSNK1G3
GSK3B
CSNK1D
APC
DVL3
BTRC
FZD5
LRP6
FZD8
AXIN2
LRP5
FZD3
ZNRF3
FZD4
LGR4
FZD6
RNF43

Figure 6

SUPPLEMENTAL INFORMATION

**Table S1. Significantly expressed miRNAs in different CSC subpopulations*.**	
LGR5/ALDH FACS Isolation	miRNAs
LGR5+/ALDH− cells	310
LGR5−/ALDH+ cells	15
CD166/ALDH FACS Isolation	miRNAs
CD166+/ALDH− cells	1
CD166−/ALDH+ cells	2
CD166/LRG5 FACS Isolation	miRNAs
CD166+/LRG5− cells	1
CD166−/LRG5+ cells	102
LRIG1/LGR5 FACS Isolation	miRNAs
LRIG1+/LGR5− cells	4
LRIG1−/LGR5+ cells	673
LRIG1/ALDH FACS Isolation	miRNAs
LRIG1+/ALDH− cells	101
LRIG1−/ALDH+ cells	4
CD166/LRIG1 FACS Isolation	miRNAs
CD166+/LRIG1− cells	29
CD166−/LRIG1+ cells	4
*Based on *p*-value < 0.05.	

Table S2. The mRNAs predicted to be targeted by the miRNAs in each CSC subpopulation were found to have different functional classifications				
LGR5 vs. ALDH	zinc finger motifs	*vs.*	phosphoproteins, and protein binding	Figure 2 vs. Figure 3
CD166 *vs*. ALDH	alternative splicing, phosphoproteins, and membrane	*vs.*	phosphoprotein, nucleus, cytoplasm, and nucleus functions	Figure S2 *vs.* Figure S3
LRIG1 *vs*. CD166	alternative splicing, transcriptional regulation, and nucleus	*vs.*	phosphoprotein functions	Figure S5 *vs.* Figure S6
LRIG1 *vs*. ALDH	alternative splicing and as protein kinase domains	*vs.*	phosphoproteins, protein binding	Figure S8 *vs.* Figure S9
LGR5 *vs*. LRIG1	cytoplasm and different protein domains	*vs.*	alternative splicing, protein binding, and phosphoprotein	Figure S11 *vs.* Figure S12
LGR5 *vs*. CD166	splice variant, phosphoprotein and zinc finger motifs	*vs.*	phosphoprotein, protein binding, cytoplasm, and nucleus	Figure S14 *vs.* Figure S15

Tables S3 & S4. BIOINFORMATICS ANALYSIS OF LGR5 and ALDH DATA

To determine what might distinguish the different CSC subpopulations from each other, a detailed analysis of the LGR5 and ALDH data was done. Then a bioinformatics analysis was done to determine which genes in the Retinoic Acid (Table 1) and WNT (Table 2) signaling pathways were predicted to be targeted by these miRNAs.

**Table S3. Retinoic Acid Pathway Genes Analyzed**	
ADH1A	Alcohol Dehydrogenase 1A
ADH1B	Alcohol Dehydrogenase 1B
ADH1C	Alcohol Dehydrogenase 1C
ADH4	Alcohol Dehydrogenase 4
ADH5	Alcohol Dehydrogenase 5
ADH6	Alcohol Dehydrogenase 6
ADH7	Alcohol Dehydrogenase 7
ALDH16A1	Aldehyde Dehydrogenase 16A1
ALDH18A1	Aldehyde Dehydrogenase 18A1
ALDH1A1	Aldehyde Dehydrogenase 1A1
ALDH1A2	Aldehyde Dehydrogenase 1A2
ALDH1A3	Aldehyde Dehydrogenase 1A3
ALDH1B1	Aldehyde Dehydrogenase 1B1
ALDH1L1	Aldehyde Dehydrogenase 1L1
ALDH1L2	Aldehyde Dehydrogenase 1L2
ALDH2	Alcohol Dehydrogenase 2
ALDH3A1	Aldehyde Dehydrogenase 3A1
ALDH3A2	Aldehyde Dehydrogenase 3A2
ALDH3B1	Aldehyde Dehydrogenase 3B1
ALDH3B2	Aldehyde Dehydrogenase 3B2
ALDH4A1	Aldehyde Dehydrogenase 4A1
ALDH5A1	Aldehyde Dehydrogenase 5A1
ALDH6A1	Aldehyde Dehydrogenase 6A1
ALDH7A1	Aldehyde Dehydrogenase 7A1
ALDH8A1	Aldehyde Dehydrogenase 8A1
ALDH9A1	Aldehyde Dehydrogenase 9A1
CRABP1	Cellular Retinoic Acid Binding Protein 1
CRABP2	Cellular Retinoic Acid Binding Protein 2
CRBP	Cellular Retinol Binding Protein
CYP26A1	Cytochrome P450 26A1
CYP26B1	Cytochrome P450 26B1
CYP26C1	Cytochrome P450 26C1
RARA	Retinoic Acid Receptor Alpha
RARB	Retinoic Acid Receptor Beta

RARG	Retinoic Acid Receptor Gamma
RBP1	Retinol Binding Protein 1
RBP2	Retinol Binding Protein 2
RBP3	Retinol Binding Protein 3
RBP4	Retinol Binding Protein 4
RBP5	Retinol Binding Protein 5
RBP7	Retinol Binding Protein 7
RDH10	Retinol Dehydrogenase 10
RDH11	Retinol Dehydrogenase 11
RDH12	Retinol Dehydrogenase 12
RDH13	Retinol Dehydrogenase 13
RDH14	Retinol Dehydrogenase 14
RDH16	Retinol Dehydrogenase 16
RDH5	Retinol Dehydrogenase 5
RDH8	Retinol Dehydrogenase 8
RXRA	Retinoid X Receptor Alpha
RXRB	Retinoid X Receptor Beta
RXRG	STRA6
STRA6	Stimulated by retinoic acid 6
TTR	Transthyretin

**Table S4. WNT Pathway Genes Analyzed**	
APC	adenomatous polyposis coli
AXIN1	aka Axin 1 a protein coding gene
AXIN2	aka Axin 2 a protein coding gene
BTRC	Beta-transducin repeat containing E3 ubiquitin protein ligase
CSNK1A1/CK1	casein kinase 1 alpha 1
CK1 beta	casein kinase 1 beta 1
CK1 gamma 1	casein kinase 1 gamma 1
CK1 gamma 2	casein kinase 1 gamma 2
CK1 gamma 3	casein kinase 1 gamma 3
CK1 delta	casein kinase 1 delta
CK1 epsilon	casein kinase 1 epsilon
CTNNB1	Beta-catenin 1
DVL1	dishevelled (DSH) DVL in mammals 1
DVL2	dishevelled (DSH) DVL in mammals 2
DVL3	dishevelled (DSH) DVL in mammals 3
FZD1	Frizzled Class Receptor 1
FZD2	Frizzled Class Receptor 2
FZD3	Frizzled Class Receptor 3

FZD4	Frizzled Class Receptor 4
FZD5	Frizzled Class Receptor 5
FZD6	Frizzled Class Receptor 6
FZD7	Frizzled Class Receptor 7
FZD8	Frizzled Class Receptor 8
FZD9	Frizzled Class Receptor 9
FZD10	Frizzled Class Receptor 10
GSK3B	Glycogen Synthase Kinase 3 Beta
LGR4	Leucine Rich Repeat Containing G Protein-Coupled Receptor 4
LGR5	Leucine Rich Repeat Containing G Protein-Coupled Receptor 5
LGR6	Leucine Rich Repeat Containing G Protein-Coupled Receptor 6
LRP5	Low-density lipoprotein receptor-related protein 5
LRP6	Low-density lipoprotein receptor-related protein 6
RNF43	Ring finger protein 43
ZNRF3	Zinc and Ring Finger 3

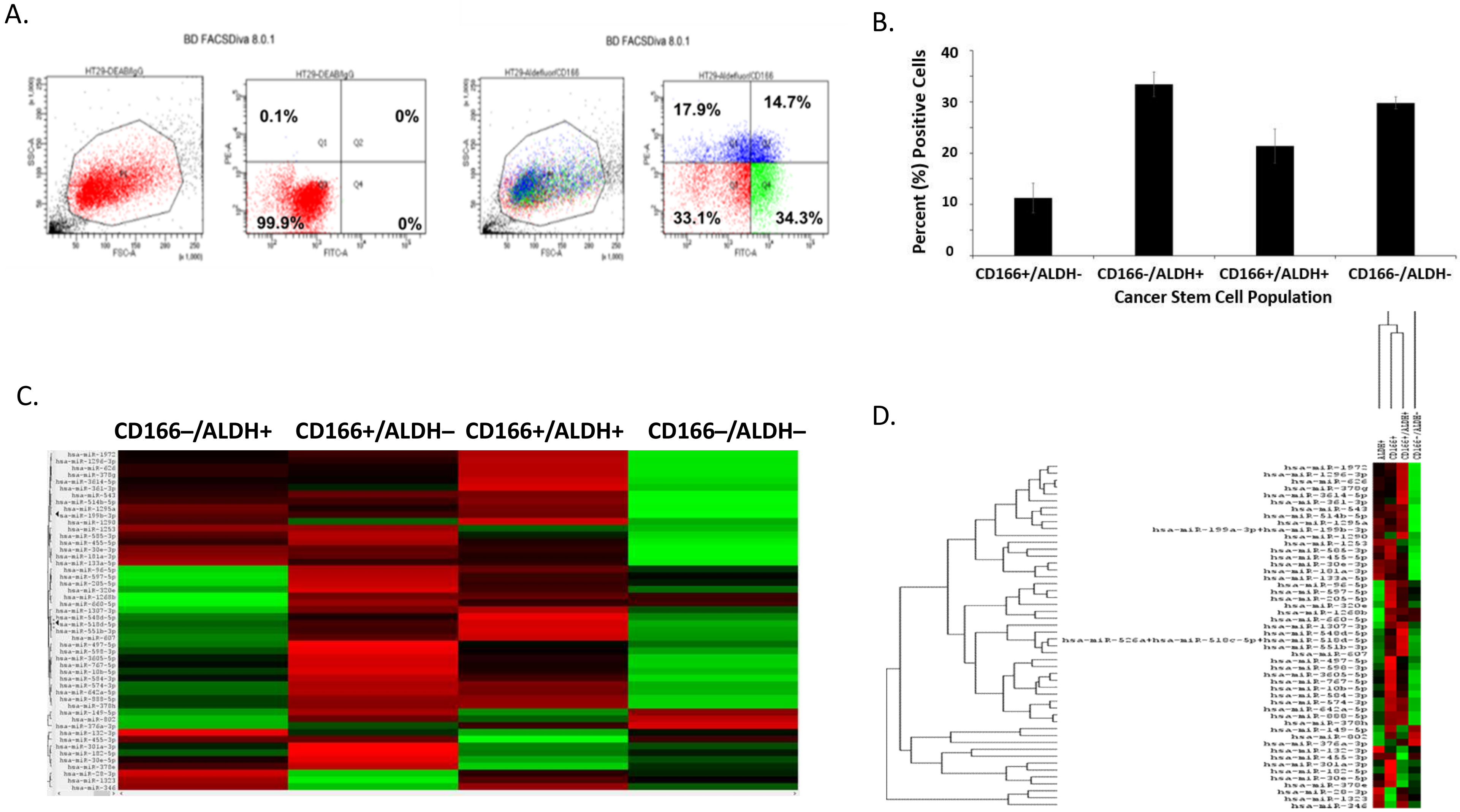

A.
BD FACSDiva 8.0.1
BD FACSDiva 8.0.1
0.1%
0%
99.9%
0%
17.9%
14.7%
33.1%
34.3%
FSC-A
SSC-A
FITC-A
PE-A
B.
Percent (%) Positive Cells
40
30
20
10
0
CD166+/ALDH-
CD166-/ALDH+
CD166+/ALDH+
CD166-/ALDH-
Cancer Stem Cell Population
C.
CD166–/ALDH+
CD166+/ALDH–
CD166+/ALDH+
CD166–/ALDH–
D.

Figure S1

E.

CD166+/ALDH- Top miRNAs

Name	*p*-Value	Ratio
hsa-miR-185-5p	0.005335	1.52
hsa-miR-34a-5p	0.079482	1.84
hsa-miR-28-5p	0.097872	1.80
hsa-miR-135b-5p	0.13191	1.42
hsa-miR-183-5p	0.148579	1.23
hsa-miR-1246	0.185359	1.91
hsa-miR-1323	0.341565	2.08
hsa-miR-26a-5p	0.377387	1.21
hsa-miR-196a-5p	0.413641	1.31
hsa-miR-1299	0.42265	1.22

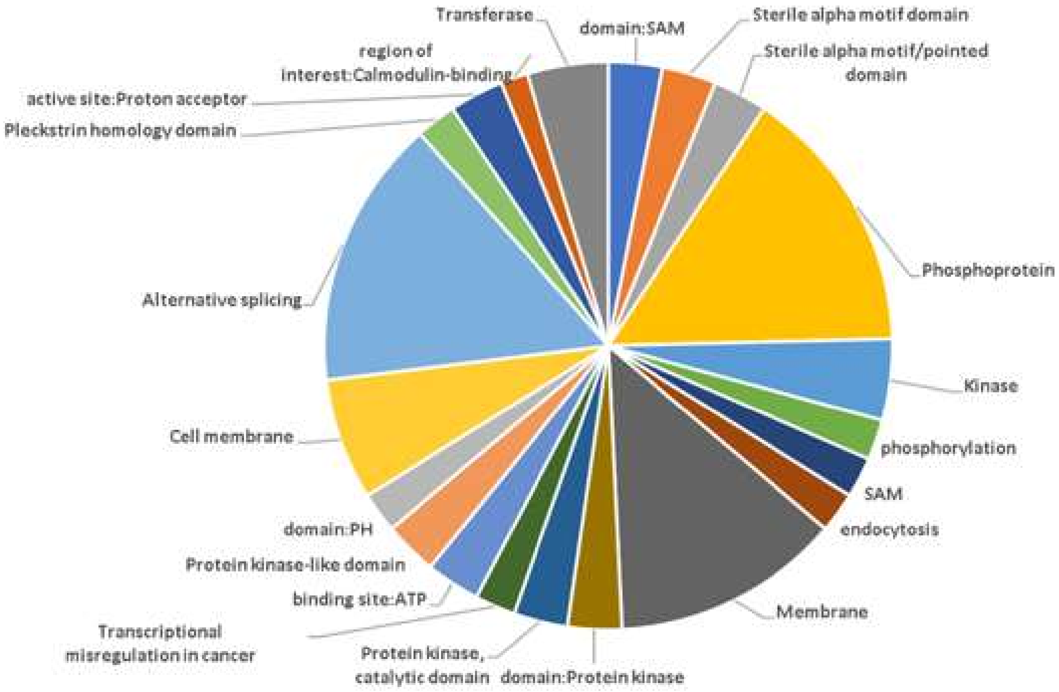

F.

ALDH+/CD166- Top miRNAs

Name	*p*-Value	Ratio
hsa-miR-301a-3p	0.024285	1.59
hsa-miR-96-5p	0.041435	1.61
hsa-miR-29b-3p	0.071412	1.18
hsa-miR-429	0.084041	1.54
hsa-miR-182-5p	0.085607	1.54
hsa-miR-660-5p	0.100104	1.92
hsa-miR-200a-3p	0.100196	1.47
hsa-miR-10a-5p	0.104863	1.16
hsa-miR-93-5p	0.106804	1.25
hsa-miR-194-5p	0.107797	1.32

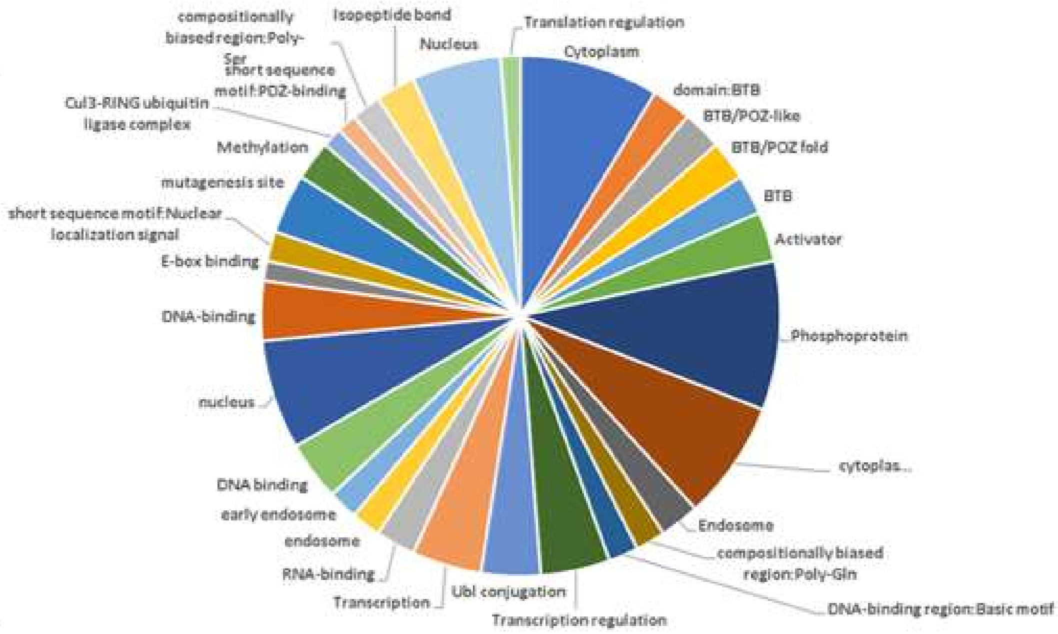

Figure S1

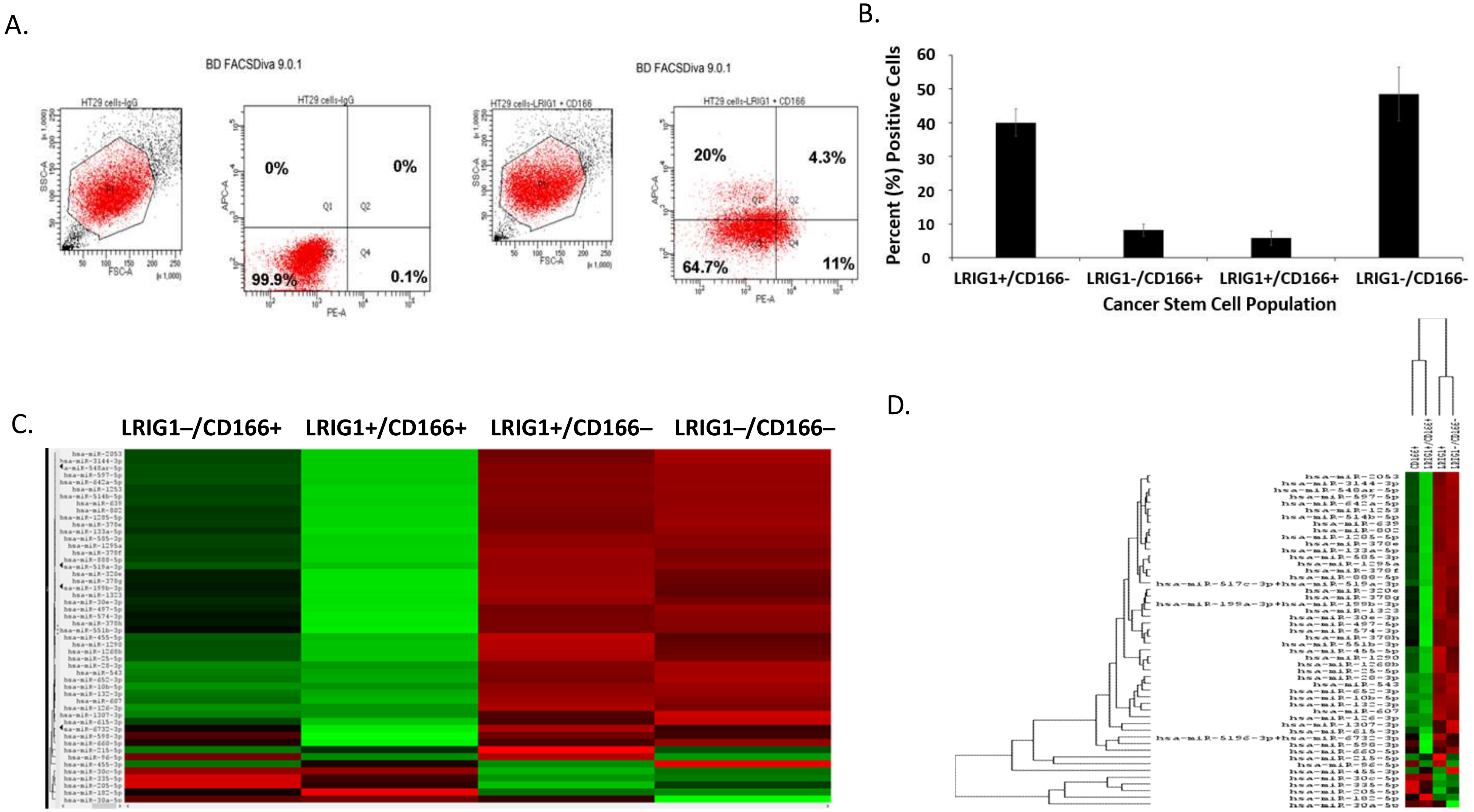
A.
BD FACSDiva 9.0.1
HT29 cells-IgG
HT29 cells-LRIG1 + CD166
SSC-A
FSC-A
APC-A
PE-A
0%
0%
99.9%
0.1%
20%
4.3%
64.7%
11%
B.
Percent (%) Positive Cells
LRIG1+/CD166-
LRIG1-/CD166+
LRIG1+/CD166+
LRIG1-/CD166-
Cancer Stem Cell Population
C.
LRIG1–/CD166+
LRIG1+/CD166+
LRIG1+/CD166–
LRIG1–/CD166–
D.

Figure S2

E.

LRIG1+/CD166- Top miRNAs

Name	*p*-Value	Ratio
hsa-miR-374b-5p	0.009738	1.39
hsa-miR-205-5p	0.010734	4.21
hsa-let-7i-5p	0.044163	1.58
hsa-miR-192-5p	0.04518	1.62
hsa-miR-429	0.0745	1.64
hsa-miR-106b-5p	0.116775	1.43
hsa-miR-590-5p	0.126675	1.56
hsa-miR-361-5p	0.128048	1.18
hsa-miR-27b-3p	0.131583	1.4
hsa-miR-374a-5p	0.198531	1.58

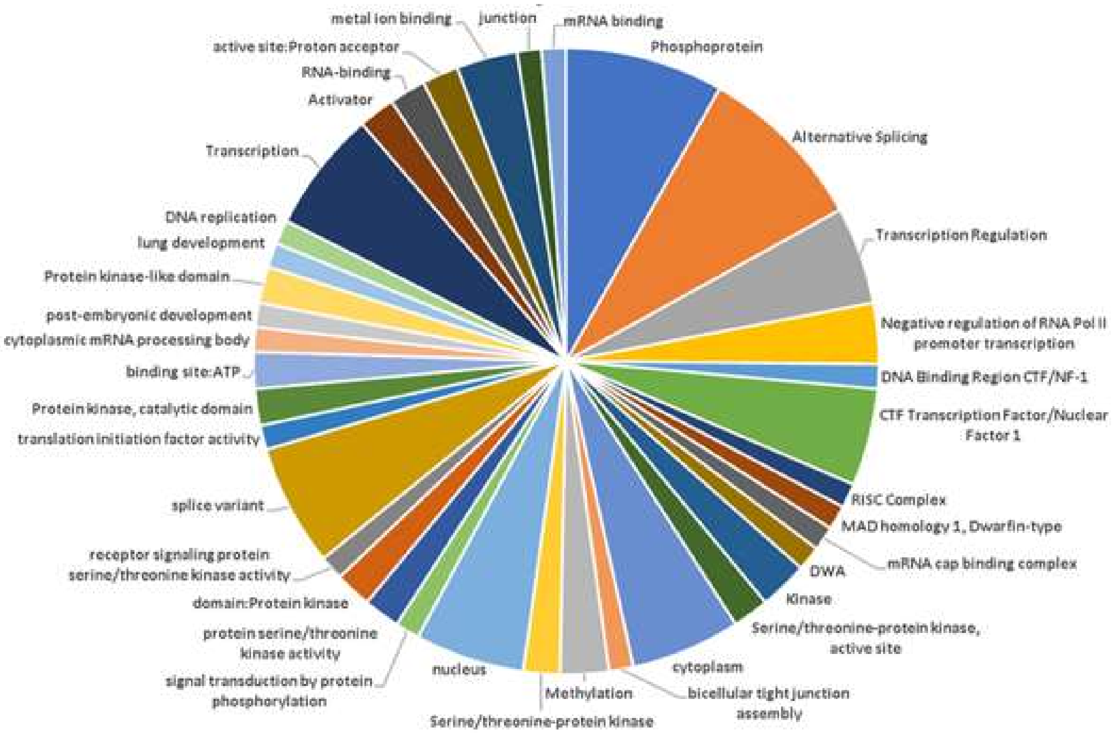

F.

CD166+/LRIG1- Top miRNAs

Name	*p*-Value	Ratio
hsa-miR-608	0.004408	1.61
hsa-miR-1200	0.006728	1.72
hsa-miR-582-3p	0.011376	2.00
hsa-miR-548y	0.012319	2.13
hsa-miR-30a-3p	0.014378	2.08
hsa-miR-28-3p	0.02381	1.64
hsa-miR-517c-3p*	0.024478	2.13
hsa-miR-1302	0.025088	2.08
hsa-miR-28-5p	0.02628	2.04
hsa-miR-628-3p	0.027493	1.75

*hsa-miR-517c-3p+hsa-miR-519a-3p

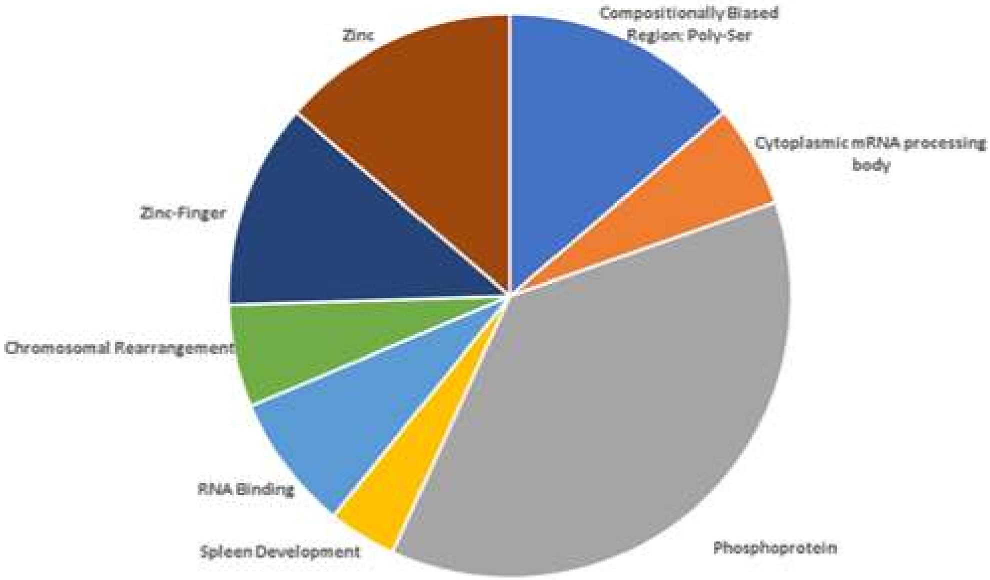

Figure S2

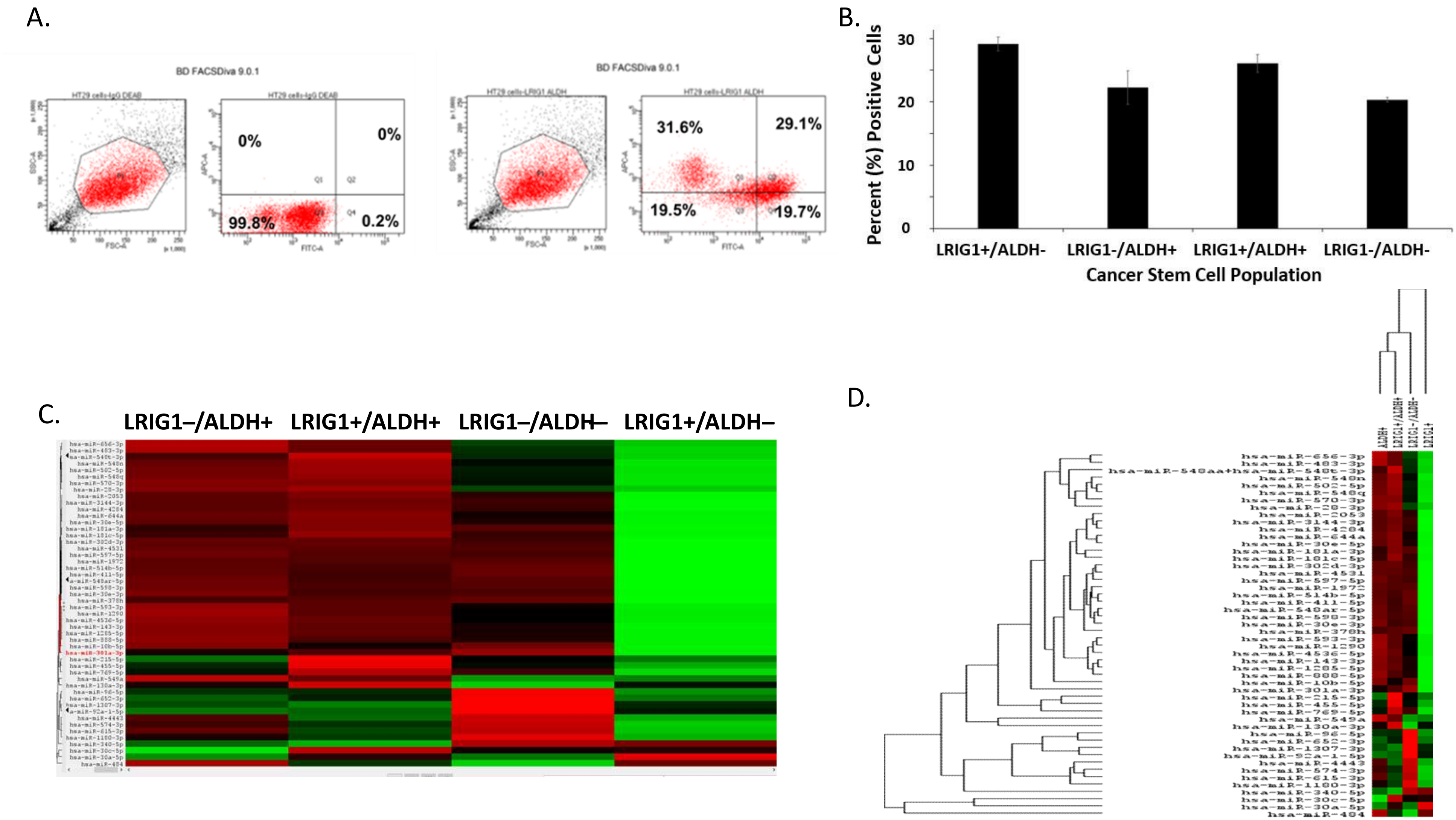

A.
BD FACSDiva 9.0.1
0%
0%
99.8%
0.2%
31.6%
29.1%
19.5%
19.7%
B.
Percent (%) Positive Cells
30
20
10
0
LRIG1+/ALDH-
LRIG1-/ALDH+
LRIG1+/ALDH+
LRIG1-/ALDH-
Cancer Stem Cell Population
C.
LRIG1–/ALDH+
LRIG1+/ALDH+
LRIG1–/ALDH–
LRIG1+/ALDH–
D.

Figure S3

E. **LRIG1+/ALDH- Top miRNAs**

Name	*p*-Value	Ratio
hsa-miR-5001-5p	0.0029	2.41
hsa-miR-193b-3p	0.007301	2.03
hsa-miR-1290	0.007807	3.22
hsa-miR-208a-3p	0.008697	3.06
hsa-miR-510-3p	0.00965	2.47
hsa-miR-365b-5p	0.010913	2.37
hsa-miR-663a	0.01348	3.70
hsa-miR-1297	0.014174	2.19
hsa-miR-205-5p	0.014201	4.33
hsa-miR-3614-5p	0.015049	3.18

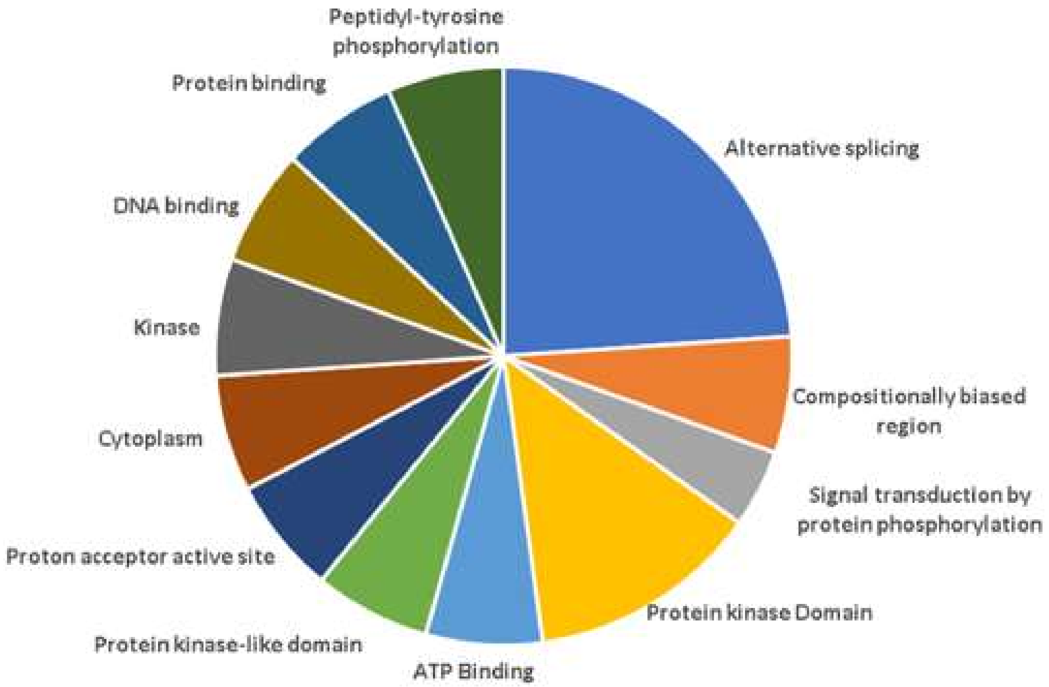

F. **ALDH+/LRIG1- Top miRNAs**

Name	*p*-Value	Ratio
hsa-miR-148a-3p	0.007872	1.64
hsa-miR-3928-3p	0.009459	1.12
hsa-miR-194-5p	0.012492	1.89
hsa-miR-375	0.021317	1.61
hsa-miR-22-3p	0.056906	1.69
hsa-miR-192-5p	0.062684	1.56
hsa-miR-185-5p	0.064618	1.32
hsa-miR-19a-3p	0.068956	1.96
hsa-miR-140-5p	0.071816	1.75
hsa-miR-27b-3p	0.078661	1.37

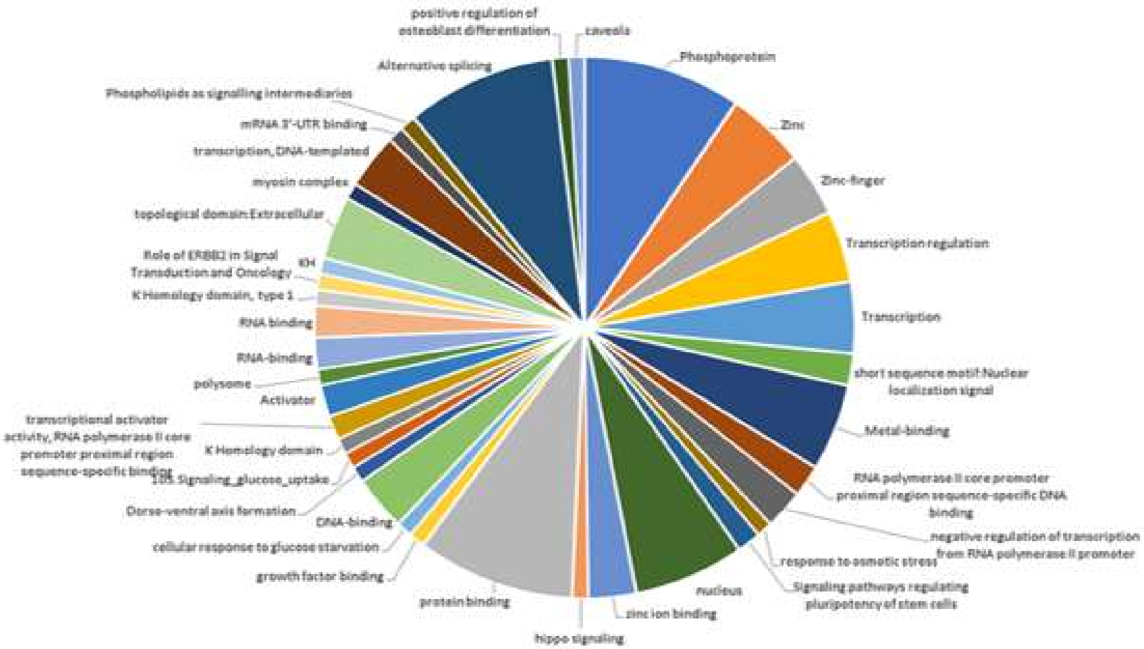

Figure S3

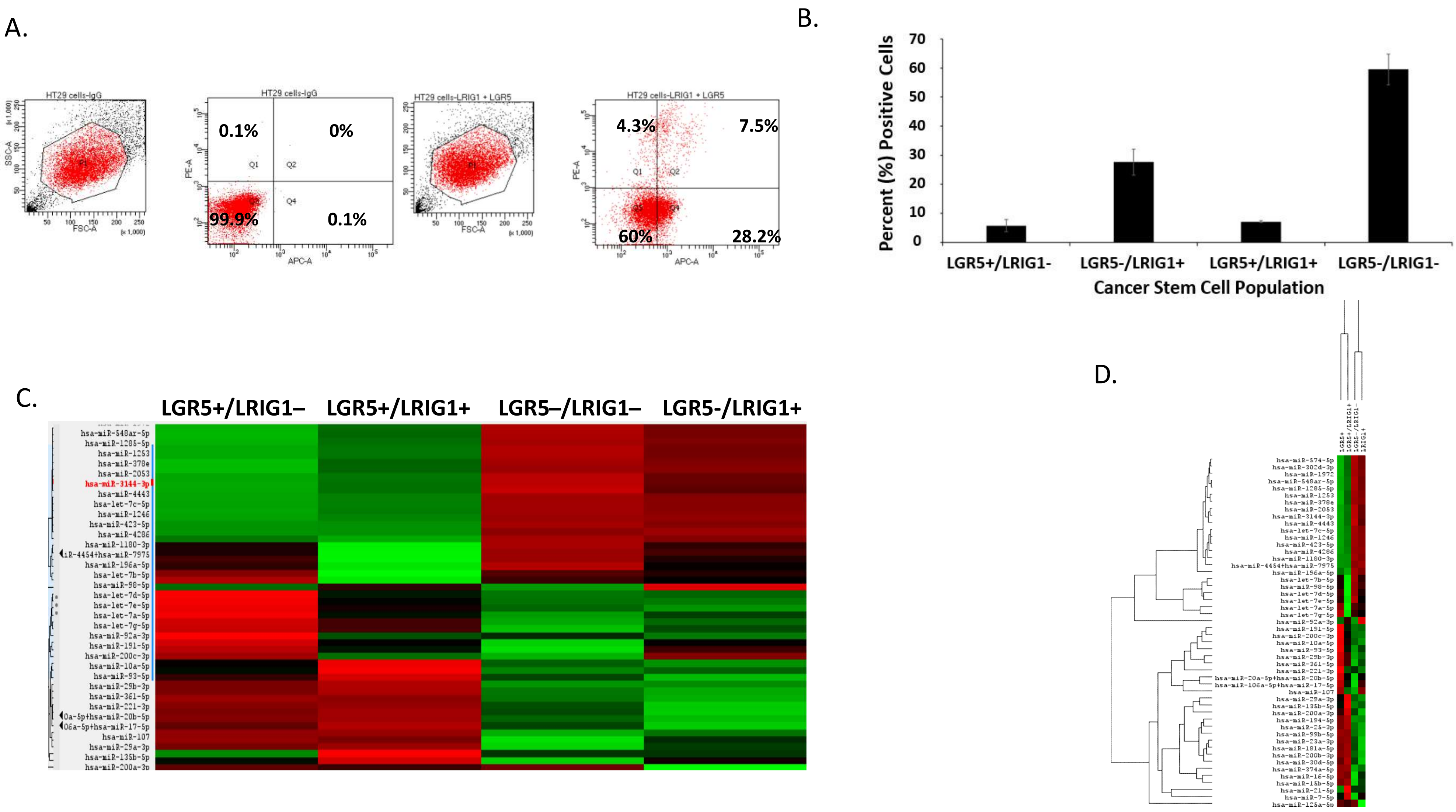
A.
B.
C.
D.
HT29 cells-IgG
HT29 cells-LRIG1 + LGR5
0.1%
0%
99.9%
0.1%
4.3%
7.5%
60%
28.2%
Percent (%) Positive Cells
Cancer Stem Cell Population
LGR5+/LRIG1-
LGR5-/LRIG1+
LGR5+/LRIG1+
LGR5-/LRIG1-
LGR5+/LRIG1–
LGR5+/LRIG1+
LGR5–/LRIG1–
LGR5-/LRIG1+

Figure S4

E.

LGR5+/LRIG1- Top miRNAs

Name	*p*-Value	Ratio
hsa-miR-455-3p	0.000516	5.626198
hsa-miR-424-5p	0.001786	7.473064
hsa-miR-454-3p	0.00185	3.375876
hsa-miR-544a	0.001903	9.050518
hsa-miR-935	0.001946	9.763292
hsa-miR-874-5p	0.002336	11.21283
hsa-miR-219a-2-3p	0.002448	13.2283
hsa-miR-218-5p	0.002587	9.866657
hsa-miR-532-5p	0.002709	6.301194
hsa-miR-1972	0.002842	24.14922

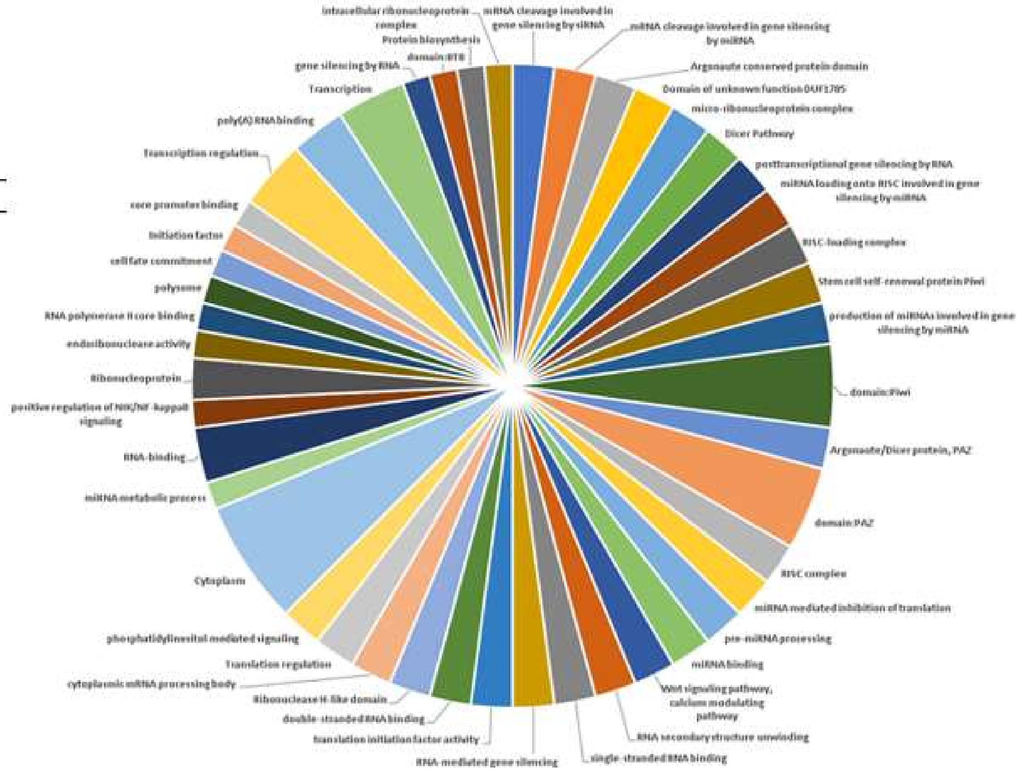

F.

LRIG1+/LGR5- Top miRNAs

Name	*p*-Value	Ratio
hsa-miR-200b-3p	0.006559	2.129456
hsa-miR-194-5p	0.010642	2.207357
hsa-miR-23a-3p	0.032224	1.784871
hsa-miR-29a-3p	0.034529	1.311528
hsa-miR-10a-5p	0.06363	2.184755
hsa-miR-30d-5p	0.071142	1.691372
hsa-miR-15b-5p	0.092976	1.587857
hsa-miR-374a-5p	0.107158	2.051671
hsa-miR-191-5p	0.120353	1.675972
hsa-miR-361-5p	0.128485	1.191644

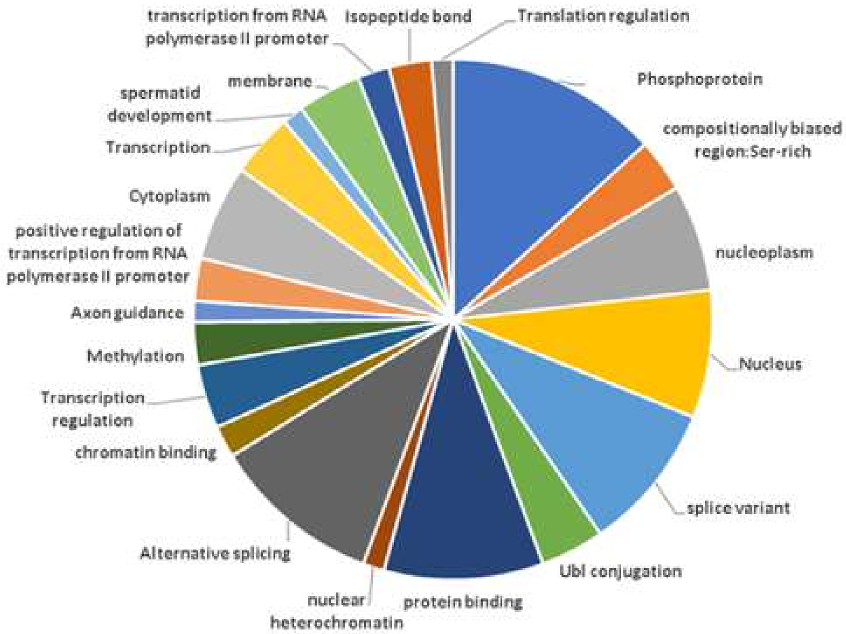

Figure S4

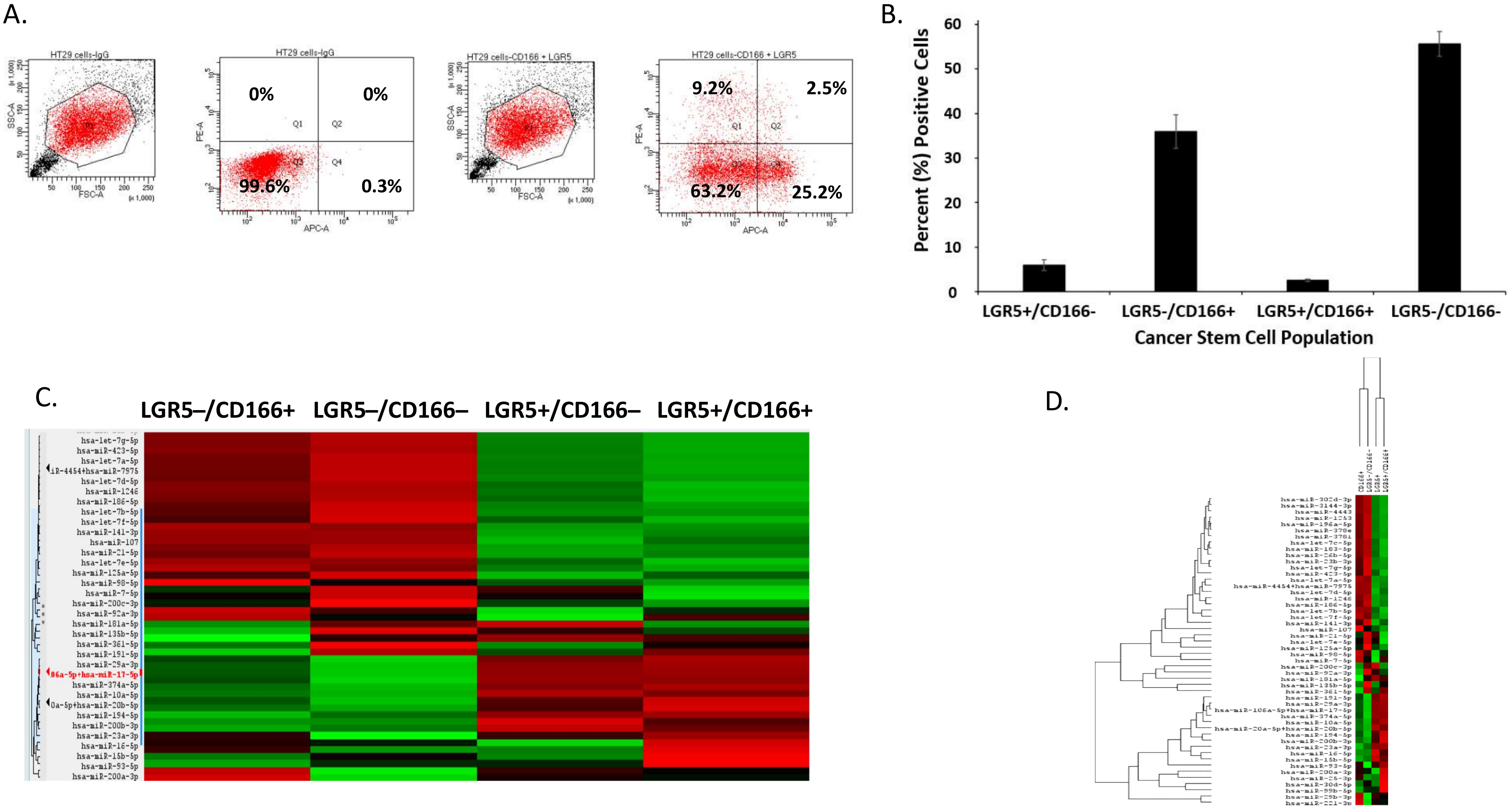
A.
HT29 cells-IgG
SSC-A
FSC-A
PE-A
APC-A
Q1
Q2
Q3
Q4
0%
0%
99.6%
0.3%
HT29 cells-CD166 + LGR5
9.2%
2.5%
63.2%
25.2%
B.
Percent (%) Positive Cells
0
10
20
30
40
50
60
LGR5+/CD166-
LGR5-/CD166+
LGR5+/CD166+
LGR5-/CD166-
Cancer Stem Cell Population
C.
LGR5–/CD166+
LGR5–/CD166–
LGR5+/CD166–
LGR5+/CD166+
hsa-let-7g-5p
hsa-miR-423-5p
hsa-let-7a-5p
hsa-let-7d-5p
hsa-miR-1246
hsa-miR-186-5p
hsa-let-7b-5p
hsa-let-7f-5p
hsa-miR-141-3p
hsa-miR-107
hsa-miR-21-5p
hsa-let-7e-5p
hsa-miR-125a-5p
hsa-miR-98-5p
hsa-miR-7-5p
hsa-miR-200c-3p
hsa-miR-92a-3p
hsa-miR-181a-5p
hsa-miR-135b-5p
hsa-miR-361-5p
hsa-miR-191-5p
hsa-miR-29a-3p
hsa-miR-374a-5p
hsa-miR-10a-5p
hsa-miR-194-5p
hsa-miR-200b-3p
hsa-miR-23a-3p
hsa-miR-16-5p
hsa-miR-15b-5p
hsa-miR-93-5p
hsa-miR-200a-3p
D.

Figure S5

E.

LGR5+/CD166- Top miRNAs

Name	*p*-Value	Ratio
hsa-miR-4454*	0.002637	8.333
hsa-miR-664b-5p	0.005766	6.667
hsa-miR-196a-5p	0.005853	1.370
hsa-miR-3918	0.010346	5.556
hsa-miR-1204	0.013975	10.000
hsa-miR-376a-2-5p	0.014634	6.250
hsa-miR-362-3p	0.01532	9.091
hsa-miR-142-5p	0.015663	5.556
hsa-miR-1908-5p	0.017958	10.000
hsa-miR-142-3p	0.019367	3.226

*hsa-miR-4454+hsa-miR-7975

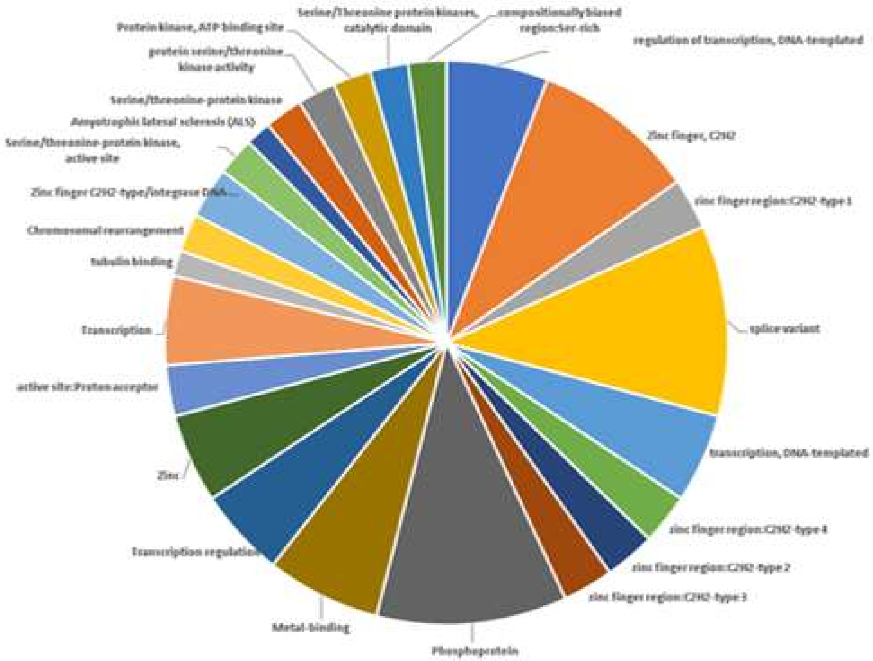

F.

CD166+/LGR5- Top miRNAs

Name	*p*-Value	Ratio
hsa-miR-15b-5p	0.034841	2.06
hsa-miR-16-5p	0.055473	2.04
hsa-miR-29a-3p	0.143658	1.41
hsa-miR-194-5p	0.165687	1.44
hsa-miR-20a-5p*	0.174565	1.48
hsa-miR-1283	0.212371	3.81
hsa-miR-181a-5p	0.213746	1.46
hsa-miR-23a-3p	0.25129	1.37
hsa-miR-106a-5p**	0.26257	1.17
hsa-let-7i-5p	0.267539	11.00

*hsa-miR-20a-5p+hsa-miR-20b-5p, **hsa-miR-106a-5p+hsa-miR-17-5p

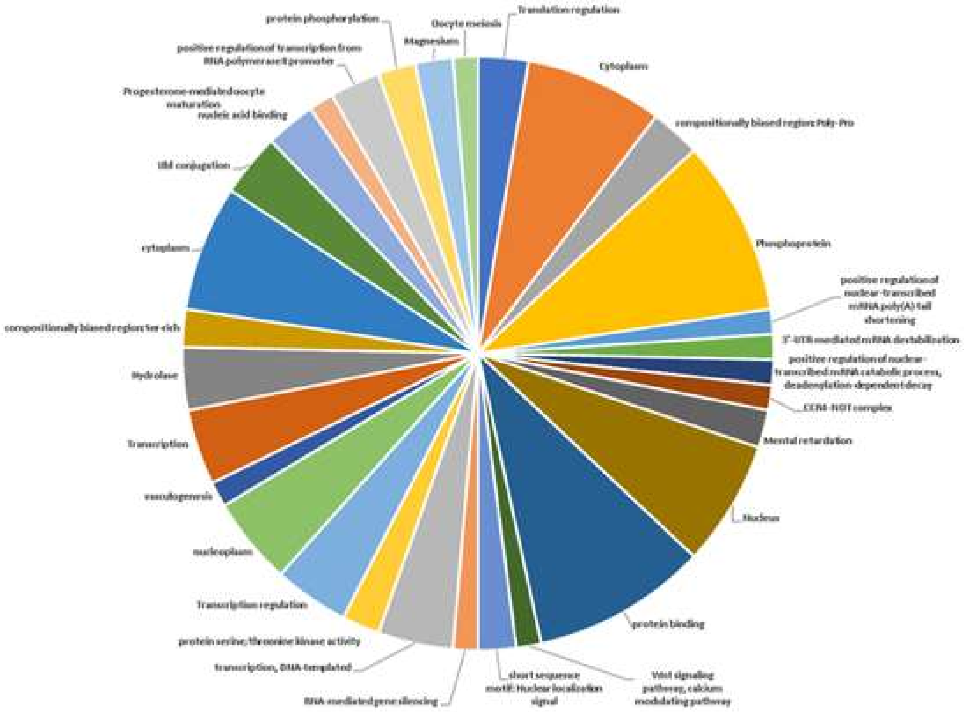

Figure S5
